# Screening for Ames mutagenicity of food flavor chemicals by (quantitative) structure-activity relationship

**DOI:** 10.1186/s41021-020-00171-1

**Published:** 2020-11-30

**Authors:** Masamitsu Honma, Airi Kitazawa, Toshio Kasamatsu, Kei-ichi Sugiyama

**Affiliations:** Division of Genetics and Mutagenesis, National Institute of Health Sciences, 3-25-26 Tonomachi, Kawasaki City, Kanagawa 210-9501 Japan

**Keywords:** Food flavors, Mutagenicity, Ames test, (quantitative) structure-activity relationship ((Q)SAR)

## Abstract

**Background:**

(Quantitative) Structure-Activity Relationship ((Q)SAR) is a promising approach to predict the potential adverse effects of chemicals based on their structure without performing toxicological studies. We evaluate the mutagenicity of food flavor chemicals by (Q) SAR tools, identify potentially mutagenic chemicals, and verify their mutagenicity by actual Ames test.

**Results:**

The Ames mutagenicity of 3942 food flavor chemicals was predicted using two (Q)SAR) tools, DEREK Nexus and CASE Ultra. Three thousand five hundred seventy-five chemicals (91%) were judged to be negative in both (Q) SAR tools, and 75 chemicals (2%) were predicted to be positive in both (Q) SAR tools. When the Ames test was conducted on ten of these positive chemicals, nine showed positive results.

**Conclusion:**

The (Q) SAR method can be used for screening the mutagenicity of food flavors.

**Supplementary Information:**

The online version contains supplementary material available at 10.1186/s41021-020-00171-1.

## Introduction

The term “food flavor” refers to a natural or synthesized chemical substance existing as an aromatic component of food. They are relatively low molecular weight chemical substances mainly composed of carbon, hydrogen, oxygen, nitrogen, and sulfur, and often have specific functional groups. In Japan, most food flavors are classified into 18 types according to their chemical structure, and currently, about 3230 individual food flavors are registered [1]. Meanwhile, about 2300 flavors and 2500 flavors are used in the United States and Europe, respectively, and approximately 1800 flavors are common to all three regions [2]. A reason behind the disparity between regions is the difference in safety assessment. Early international harmonization is desired.

Even though in daily use, up to hundreds of flavorings are mixed and used, the amount of each flavor added to food is in the order of the ppb or ppm level. Therefore, there is no concern about the general toxicity of these substances, because many substances have a level below which toxic effects are not observed, i.e., a threshold [3]. However, regarding the mutagenicity, health concerns arise even with minute amounts. Mutagenic chemicals damage DNA, induce mutations, and cause cancer [4]. Mutagenicity is considered to have no threshold [3]. Since carcinogenic risk never reaches zero, the intake of mutagenic chemicals requires strict control, even at extremely low amounts. FAO/WHO Joint Expert Committee on Food Additives (JECFA) states that any toxicity, including mutagenicity, is not a concern if the exposure level is below the threshold of toxicity concern (TTC) [5]. However. the presence or absence of mutagenicity often poses a problem if the exposure assessment is not carried out appropriately. Therefore, evaluating the safety of food flavors require relevant mutagenicity tests.

The bacterial reverse mutation test (Ames test) is an important mutagenicity test, but it requires approximately 2 g of sample for a dose-finding study and main study [6]. On the other hand, the amount of flavor produced industrially is extremely small, often rendering testing impossible. Besides, the peculiar odor of some flavors sometimes makes it difficult to perform the test in the laboratory. Therefore, an effective screening tool that promptly and accurately evaluates the mutagenicity of flavors without performing an Ames test is required.

(Quantitative) Structure-Activity Relationship ((Q)SAR) is a promising approach to predict the potential adverse effects of chemicals based on their structure without performing toxicological studies [7]. Much effort has been invested in the development of (Q) SAR tools to predict Ames mutagenicity (among other toxicological endpoints) because of the large amounts of Ames test data that have already been accumulated [8]. The International Council for Harmonization of Pharmaceutical Regulations (ICH) M7 guideline, “Assessment and control of DNA reactive (mutagenic) impurities in pharmaceuticals to limit potential carcinogenic risk” approved (Q) SAR methodologies as an alternative to the Ames test to evaluate the mutagenicity of impurities [9]. These guidelines increased the interest in using (Q) SARs for human health assessments in other areas. Many (Q) SAR tools have been used for evaluating the mutagenicity of pharmaceutical candidates, food chemicals, industrial chemicals, environmental pollutants, etc. [10–13]. The ICH-M7 guideline recommends the use of two (Q) SAR tools that complement each other, one rule-based and one statistical-based, to evaluate the Ames mutagenicity, [10]. In the present study, we evaluate the mutagenicity of 3942 food flavor chemicals used all over the world by two (Q) SAR tools according to ICH-M7 guideline, identify potentially mutagenic chemicals, and verify their mutagenicity by actual Ames test.

## Materials and methods

### Database of food flavor chemicals for (Q) SAR analysis

The database of flavor chemicals (2015) was obtained from the Japan Flavor & Fragrance materials Association (JFFMA). It lists 4549 flavor chemicals. Of the 4549 chemical substances, 3942 single-molecule flavor chemicals were re-listed for (Q) SAR analysis, excluding 607 compounds that are mixtures or have no structural formula.

### (Q) SAR tools

Derek Nexus is a knowledge-based commercial software developed by Lhasa Limited, UK [14, 15]. It includes knowledge rules created considering insights related to structural alert (SA), chemical compound examples, and metabolic activations and mechanisms. These knowledge rules are continuously developed by the provision of data and knowledge by private corporations, universities, public research institutions, and nonprofit organizations. Derek Nexus compares the query chemical to toxicophores (that is, a structure that is assumed to be involved in mutagenicity) encoded in the knowledge base. If the query triggers an SA, Derek Nexus ranks the possibility of mutagenicity (certain, probable, plausible, equivocal, doubted, improbable, impossible, open, contradicted, nothing to report) by applying a “reasoning rule”. When it is “certain”, “probable”, “plausible”, or “equivocal”, the query chemical is predicted as positive in the Ames test. Derek Nexus is updated about once a year, and version 6.0.1 (which has 117 SA for Ames mutagenicity) was used in this study.

CASE Ultra is a QSAR-based toxicity prediction software developed by MultiCASE Inc., US. CASE Ultra uses a statistical method to automatically extract alerts based on training data using machine learning technology. The data required for training are the chemical structures and their toxicity labels [16, 17]. The degree of toxicity predicted for the queried chemical substance depends not only on the specified alert but also on the structural environment around the alert. The structural characteristics of the alert surroundings are called the “modulator,” and this is also learned automatically from the training data. In this algorithm, to construct a QSAR model with continuous toxicity endpoints, various physical chemistry parameters and descriptors are used. In this study, we used CASE Ultra version 1.6.2.1 with five modules related to Ames mutagenicity: GT1_A7B, GT1_AT_ECOLI, PHARM_SALM, PHARM_ECOLI, and GT_EXPERT. The prediction result of each module is ranked as “known positive,” “positive,” “negative,” “known negative,” “inconclusive,” or “out of domain.” A query chemical ranked “known positive” or “positive” in at least one module is predicted as positive in the Ames test.

### Ames tests

Ames tests were conducted for ten flavor chemicals. The chemicals purities and suppliers are shown in Table 1. The Ames tests were conducted by contract research organizations under GLP compliance according to the Industrial Safety and Health Act test guideline [18]. The test guideline requires five stains (S. thyphimurium TA100, TA98, TA1535, TA1537, and *E. coli* WP2 uvrA) under both the presence and absence of the metabolic activation (rat S9mix), which is similar to the OECD guideline TG471 [19]. The positive criterion is when the number of revertant colonies increased more than twice as much as the control in at least one Ames test strain in the presence or absence of S9mix. Dose-dependency and reproducibility were also considered in the final judgment. A chemical with more than 1000 revertant colonies per mg (Relative Activity Value; RAV) was considered strongly positive.

**Table 1:** Flavor chemicals in which Ames test was newly conducted.

No.	JECFA No.	Chemical Name	CAS No.	Purity (%)	Supplier	Category^a^
I	470	2-[(methylthio)methyl]-2-butenal	40878-72-6	98.1	T. HASEGAWA CO.,LTD.	Aliphatic higher aldehydes
II	687	4'-methoxycinnamaldehyde	1963-36-6	98	Alfa Aesar	Aromatic aldehydes
III	1208	4-methyl-2-pentenal	5362-56-1	99.2	T. HASEGAWA CO., LTD.	Aliphatic higher aldehydes
IV	1451	4-methoxy-2,5-dimethyl-3(2H)-furanone	4077-47-8	97	Tokyo Chemical Industry Co.,Ltd.	Ketones
V	1456	2,5-dimethyl-4-oxo-3(5H)-furyl acetate	4166-20-5	>95	Takata Koryo Co., Ltd.	Esters
VI	1506	3-acetyl-2,5-dimethylfuran	10599-70-9	98	Tokyo Chemical Industry Co., Ltd.	Ketones
VII	2101	furfuryl formate	13493-97-5	>98.9	T. HASEGAWA CO., LTD.	Esters
VIII	2144	methyl beta-phenylglycidate	37161-74-3	99.8	T. HASEGAWA CO., LTD.	Esters
IX	2157	6-methoxyquinoline	5263-87-6	98.9	Tokyo Chemical Industry Co., Ltd.	Ethers
X	-	2-methylquinoline	91-63-4	98	Tokyo Chemical Industry Co., Ltd.	Not classified ^b^

^a^ Eighteen categories (and other than specified else) classified according to their substructures defined in the Japanese Food Sanitation Law

^b^ Not applicable to flavor chemical in Japan

## Results

**Fig. 1:**
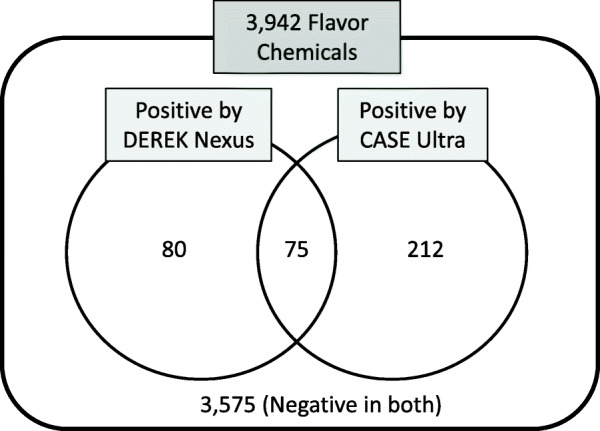
Results of QSAR prediction for Ames mutagenicity by DEREK Nexus and CASE Ultra

We used two (Q) SAR tools (DEREK Nexus and CASE Ultra) to predict the Ames mutagenicity of 3942 newly listed single-molecule flavor chemicals. Of these, 155 were predicted positive (equivocal, plausible, probable) by DEREK Nexus, and 287 were predicted positive (positive, known positive) by at least one of the five modules of CASE Ultra. Seventy-five chemicals were predicted as positive by both (Q) SAR tools (2%) and were therefore considered highly probably mutagenic, and 3575 chemicals (91%) were judged negative in both (Q) SAR tools (Fig. 1).

To verify the predicted positive results, we conducted Ames tests for these chemicals. We excluded 22 chemicals, for which the Ames test’s results were already reported [20]. We then selected ten chemicals (based on availability) and carried out the Ames tests (Table 2). The results are shown in Additional file 1. Nine out of ten chemicals exhibited positive Ames test results (Table 2). In particular, three chemicals, 4-methyl-2-pentenal (III), 3-acetyl-2,5-dimethylfuran (VI), and 6-methoxyquinoline (IX) showed a strong positive response with a RAV of 1000 or more. One chemical, 4′-methoxycinnamaldehyde (II) displayed a weak response in TA100 in the presence of S9mix. Although the number of revertant colonies was less than twice that of the control, the weak response was dose-dependent and reproducible.

**Table 2:**
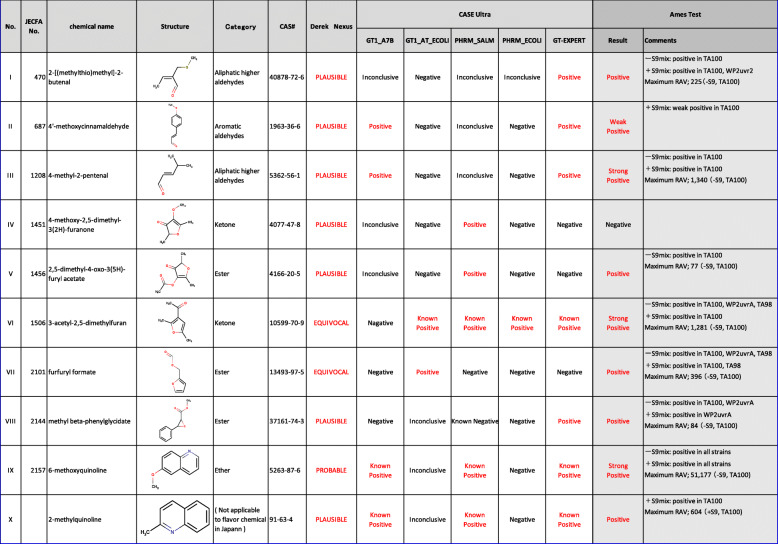
The results of QSAR prediction and Ames tests of 10 flavor chemicals.

## Discussion

The ICH recently released the ICH-M7 guideline for the assessment and control of mutagenic impurities in pharmaceuticals to limit potential carcinogenic risk [9]. This guideline allows the use of (Q) SAR tools to predict Ames mutagenicity for the initial assessment of impurities in pharmaceuticals. This approach is sensible because pharmaceuticals usually have very low levels of impurities, which makes them tricky to isolate and purify for the Ames test. The Ames mutagenicity test for flavor chemicals is also challenging in most cases. This is due to the very low production amounts, which makes it difficult to obtain enough chemical to conduct the toxicological study, and the strong smell, which makes conducting the actual test in the laboratory laborious. Consequently, the safety of many flavor chemicals has not been accurately assessed. We expect that the application of (Q) SAR to the evaluation of the mutagenicity of flavor chemicals will solve these problems.

Ono et al. [21] assessed the viability of (Q) SAR tools in 2012 by calculating the Ames mutagenicity of 367 flavor chemicals (for which Ames test results were available) using three (Q) SAR tools including Derek for Windows and MultiCASE, which are old models of Derek Nexus and CASE Ultra, respectively. All three (Q) SAR tools had a low (14–39%) sensitivity (the ability of a (Q) SAR tool to detect Ames positives chemicals correctly) as well as low (32–39%) positive prediction value (the frequency of correct positive predictions). These results implied that the application of (Q) SAR tools to assess the Ames mutagenicity of flavor chemicals was still premature. In recent years, however, the prediction power of Ames (Q) SAR tools has improved [8]. Honma et al. conducted the Ames/(Q) SAR International Challenge Project with 12 (Q) SAR vendors to validate and improve the Ames mutagenicity prediction of their tools from 2014 to 2017 [20]. A large new Ames test database (12,140 chemicals) in his institute improved the prediction power of (Q) SAR tools, leading to the success of the project. Lhasa limited and MultiCASE Inc. also participated in this project, and Derek Nexus and CASE Ultra used this study to obtain a higher prediction power than with their old models in 2012 [20].

The ICH-M7 guideline recommends the use of two (Q) SAR tools that complement each other, one rule-based and one statistical-based, to evaluate the Ames mutagenicity, and mentions that a negative prediction by both (Q) SAR tools should lead to the conclusion that there is no mutagenic concern [9]. We initially used Derek Nexus version 6.0.1 as a rule-based SAR tool and CASE Ultra version 1.6.2.1 as a statistical-based QSAR tool. However, CASE Ultra version 1.6.2.1 is equipped with both statistical-based modules and a rule-based module (GT_EXPERT). This may be the reason why CASE Ultra yielded more positive flavor chemicals than Derek Nexus (Fig. 1). Therefore, the screening in this study can be regarded as a combination of two rule-based SARs and one statistical-based QSAR, which is more conservative than what the ICH-M7 recommends. This approach excluded 3575 flavor chemicals (91%) as non-mutagens (Fig. 1) which is consistent with the report by Honma et al. that about 85% of new industrial chemicals are negative in the Ames test [17]. Considering the properties of flavor chemicals (low molecular weight, limited elements, limited chemical structure), they are less likely to be mutagenic than general industrial chemicals. Therefore, the absence of mutagenicity in 91% of flavors is credible and shows the relevance of combined (Q) SAR tools to screen the Ames mutagenicity of flavor chemicals.

On the other hand, there are concerns about the mutagenicity of the remaining 367 chemicals (9%). According to ICH-M7, if a chemical is predicted to have potential of mutagenicity by at least one of the two (Q) SAR tools, it is considered a mutagen. Therefore, to verify the mutagenicity of these 367 chemicals, it is necessary to actually conduct the Ames test. Of these, 75 chemicals were predicted to be positive in both (Q) SAR tools, which are likely to be mutagenic in the Ames test. Of these, ten chemicals were selected, and the Ames tests were conducted, and nine chemicals were positive (90%), indicating that the combination of the (Q) SAR tools can accurately identify mutagenic flavor chemicals.

After the Ames test, we re-calculated the Ames mutagenicity of the ten chemicals by the latest version of Derek Nexus (version 6.1.0) and CASE Ultra (version 1.8.0.2). The only statistical-based module in this version of CASE Ultra is GT1_BMUT. The results obtained by Derek Nexus were the same as those of the previous version, while CASE Ultra 1.8.0.2 gave negative predictions for 4-methoxy-2,5-dimethyl-3(2H)-furanone (IV) and 2,5-dimethyl-4-oxo-3(5H)-furyl acetate (V), and inconclusive for furfuryl formate (VII). These three chemicals have a furan structure, and their mutagenic mechanism remains unclear. The mutagenic mechanisms of the ten chemicals tested by the Ames test are discussed below.

2-[(methylthio) methyl] -2-butenal (I) and 4-methyl-2-pentenal (III), which are aliphatic higher aldehydes containingα, β-unsaturated carbonyl, were positive in TA100 both in presence and absence of S9mix. 4-methyl-2-pentenal (III) showed strong mutagenicity with 1340 RAV in TA100 in the absence of S9mix. α, β-unsaturated carbonyls are bis electrophiles that can react with electron-rich biological macromolecules. In addition to the carbon of the carbonyl functional group, the β-carbon is positively polarized due to the conjugation with the carbonyl group, making it a preferential site for a nucleophilic attack like the classical Michael-type addition [22]. In addition to their common structural features, theseα, β-unsaturated carbonyls can each interact with DNA in their own way, causing different genotoxic and mutagenic reactions possibly via circular adduct formation, frameshift mutations, strand breaks, and cross-linking. Besides direct interactions, other metabolic activation pathways such as metabolic epoxidation and radical formation can occur [7, 23].

4′-methoxycinnamaldehyde (II) is an aromatic aldehyde and hasα, β-unsaturated carbonyl structure. Because it showed weak mutagenicity only in the presence of S9mix, however, its mutagenic mechanism is expected to be different from that of the previous chemicals (I, III). It may be converted to a sulfate conjugate by a sulfate transferase, (a Phase II enzyme), and then the sulfate group may dissociate to generate carbonium ions [24]. Therefore, it should be noted that its mutagenicity may be more pronounced in vivo where the Phase II enzymes are more active [25].

2,5-dimethyl-4-oxo-3(5H)-furyl acetate (V) and furfuryl formate (VII) are classified as esters, but their mutagenic activity is expected to involve double bond epoxidation. The ester methyl beta-phenylglycidate (VIII) also has an epoxy structure and is expected to display the same mutagenic mechanism. These chemicals exert their mutagenicity without metabolic activation. 3-acetyl-2,5-dimethylfuran (VI), which is classified as a ketone but has a furan structure, showed up as a strong positive without S9mix. Notably, 4-methoxy-2,5-dimethyl-3(2H)-furanone (IV) was the only negative in this study. This chemical also has a furan structure and is quite similar to 2,5-dimethyl-4-oxo-3(5H)-furyl acetate (V). The only differences are the acetate group and the methoxy group. This result is inconsistent because the methoxy group is generally considered a mutagenicity-enhancing group [26]. Further studies are needed to understand the mutagenic mechanisms of furan flavors.

6-methoxyquinoline (IX) and 2-methylquinoline (X) showed a strong positive and positive response, respectively, in the presence of S9mix. The main metabolic pathway for their mutagenicity is 3,4-epoxidation by metabolic activation [7]. As a result, the ring-opening metabolite exerts its mutagenicity by covalently binding to DNA. 6-methoxyquinoline (IX) is expected to be more mutagenic than quinoline because of the methoxy group in position 6, which is a mutagenicity-enhancing group [26]. Given that the methyl group of 2-methylquinoline (X) is also mutagenicity-enhancing, its mutagenicity is expected to be between 6-methoxyquinoline (IX) and quinoline.

## Conclusion

We screened Ames mutagenicity of 3942 food flavor chemicals using two (Q) SAR tools. We excluded 3575 flavor chemicals (91%) from the potential health hazards list and selected 75 flavor chemicals (2%) which are highly suspected of mutagenicity. We then verified the mutagenicity of ten selected chemicals by performing an actual Ames test and found that nine of these ten chemicals (90%) came out positive, indicating that (Q) SAR screening is a powerful tool for the safety assessment of food flavors. Food flavors showing mutagenicity in (Q) SARs or Ames tests may present a risk of carcinogenicity in humans even when used in minute quantities. Further assessment of in vivo mutagenicity is required.

## Supplementary Information

**Additional file 1.**

## Data Availability

All generated data are included in this manuscript. Raw data for the Ames tests are available in the Appendix.

## Abbreviations

(Q)SAR: (Quantitative) structure-activity relationship
ICH: International Council for Harmonisation of Technical Requirements for Pharmaceuticals for Human Use
JECFA: Joint FAO/WHO Expert Committee on Food Additives
TTC: Threshold of Toxicity Concern
JFFMA: Japan Flavor & Fragrance materials Association
