# Supplementary material for: Screening for Ames mutagenicity of food flavor chemicals by (quantitative) structure-activity relationship
Source: Genes Environ. 2020 Nov 30;42:32. doi: 10.1186/s41021-020-00171-1 (PMC7706032; doi:10.1186/s41021-020-00171-1)
Supplement: Supplementary file 1 — Additional file 1. [file 41021_2020_171_MOESM1_ESM.docx]

| Metabolic  activation | | Dose  (µg/plate) | Colonies /plate | | | | |
| --- | --- | --- | --- | --- | --- | --- | --- |
|  |  |  | Base-substitution | | | Frameshift | |
|  |  |  | TA100 | TA1535 | WP2 *uvrA* | TA100 | TA1537 |
| S9 mix  (-) | | Negtive control | 113  107 ( 110 ) | 11  13 ( 12 ) | 31  40 ( 36 ) | 12  18 ( 15 ) | 14  7 ( 11 ) |
|  |  | 5.00 | 102  109 ( 106 ) | 5  11 ( 8 ) | 35  33 ( 34 ) | 7  17 ( 12 ) | 4  13 ( 9 ) |
|  |  | 15.0 | 106  113 ( 110 ) | 13  5 ( 9 ) | 24  49 ( 37 ) | 17  18 ( 18 ) | 14  16 ( 15 ) |
|  |  | 50.0 | 97  114 ( 106 ) | 9  6 ( 8 ) | 37  35 ( 36 ) | 14  16 ( 15 ) | 14  13 ( 14 ) |
|  |  | 150 | 135  115 ( 125 ) | 4  11 ( 8 ) | 29  41 ( 35 ) | 19  11 ( 15 ) | 7  9 ( 8 ) |
|  |  | 500 | 214  169 ( 192 ) | 9  13 ( 11 ) | 33  30 ( 32 ) | 13  18 ( 16 ) | 10  19 ( 15 ) |
|  |  | 1500 | 395  452 ( 424 ) | 16  12 ( 14 ) | 44  41 ( 43 ) | 20  21 ( 21 ) | 11  8 ( 10 ) |
|  |  | 5000 | 0 *  0 * ( 0 ) | 0 *  0 * ( 0 ) | 68 *  63 * ( 66 ) | 0 *  0 * ( 0 ) | 0 *  0 * ( 0 ) |
| S9 mix  (+) | | Negtive control | 131  123 ( 127 ) | 9  10 ( 10 ) | 48  45 ( 47 ) | 20  20 ( 20 ) | 21  16 ( 19 ) |
|  |  | 5.00 | 132  114 ( 123 ) | 12  14 ( 13 ) | 47  48 ( 48 ) | 33  22 ( 28 ) | 21  25 ( 23 ) |
|  |  | 15.0 | 120  110 ( 115 ) | 10  7 ( 9 ) | 43  38 ( 41 ) | 34  25 ( 30 ) | 17  23 ( 20 ) |
|  |  | 50.0 | 129  113 ( 121 ) | 9  12 ( 11 ) | 46  34 ( 40 ) | 19  22 ( 21 ) | 21  24 ( 23 ) |
|  |  | 150 | 107  123 ( 115 ) | 12  9 ( 11 ) | 35  36 ( 36 ) | 22  26 ( 24 ) | 27  18 ( 23 ) |
|  |  | 500 | 181  154 ( 168 ) | 8  13 ( 11 ) | 44  46 ( 45 ) | 23  29 ( 26 ) | 24  15 ( 20 ) |
|  |  | 1500 | 301  296 ( 299 ) | 10  16 ( 13 ) | 40  37 ( 39 ) | 21  30 ( 26 ) | 13  14 ( 14 ) |
|  |  | 5000 | 0 *  0 * ( 0 ) | 19 *  14 * ( 17 ) | 91  69 ( 80 ) | 0 *  0 * ( 0 ) | 0 *  5 * ( 3 ) |
|  | S9 mix  (-) | Positive controls | AF-2 | SA | AF-2 | AF-2 | 9AA |
|  |  | Dose (µg/plate) | 0.01 | 0.5 | 0.01 | 0.1 | 80 |
|  |  | Colonies/plate | 408  426 ( 417 ) | 561  581 ( 571 ) | 158  148 ( 153 ) | 573  534 ( 554 ) | 563  545 ( 554 ) |
|  | S9 mix  (+) | Positive controls | B[a]P | 2AA | 2AA | B[a]P | B[a]P |
|  |  | Dose (µg/plate) | 5 | 2 | 10 | 5 | 5 |
|  |  | Colonies/plate | 1325  1275 ( 1300 ) | 476  529 ( 503 ) | 690  761 ( 726 ) | 409  470 ( 440 ) | 168  160 ( 164 ) |

**Appendix Ia: 2-[(methylthio)methyl]-2-butenal (Exp. 1)**

( ), the average number of colonies. Negative control, DMSO

AF-2, 2-(2-Furyl)-3-(5-nitro-2-furyl)acrylamide; SA, Sodium azide; 9AA, 9-Aminoacridine; B[a]P, Benzo[a]pyrene; 2AA, 2-Aminoanthracene

†, Precipitation

*, Growth inhibition

**Appendix Ib: 2-[(methylthio)methyl]-2-butenal (Exp. 2)**

| Metabolic  activation | | Dose  (µg/plate) | Colonies /plate | | | | |
| --- | --- | --- | --- | --- | --- | --- | --- |
|  |  |  | Base-substitution | | | Frameshift | |
|  |  |  | TA100 | TA1535 | WP2 *uvrA* | TA98 | TA1537 |
| S9 mix  (-) | | Negtive control | 116  117 ( 117 ) | 13  10 ( 12 ) | 26  30 ( 28 ) | 11  19 ( 15 ) | 16  16 ( 16 ) |
|  |  | 156 | 133  125 ( 129 ) | 10  14 ( 12 ) | 29  21 ( 25 ) | 18  12 ( 15 ) | 10  17 ( 14 ) |
|  |  | 313 | 163  168 ( 166 ) | 15  5 ( 10 ) | 30  25 ( 28 ) | 23  18 ( 21 ) | 22  12 ( 17 ) |
|  |  | 625 | 229  237 ( 233 ) | 9  19 ( 14 ) | 25  25 ( 25 ) | 16  18 ( 17 ) | 14  14 ( 14 ) |
|  |  | 1250 | 426  370 ( 398 ) | 10  13 ( 12 ) | 26  36 ( 31 ) | 27  23 ( 25 ) | 14  22 ( 18 ) |
|  |  | 2500 | 383  401 ( 392 ) | 7  14 ( 11 ) | 58  34 ( 46 ) | 26  37 ( 32 ) | 11  11 ( 11 ) |
|  |  | 5000 | 0 *  0 * ( 0 ) | 0 *  0 * ( 0 ) | 50 *  53 * ( 52 ) | 0 *  0 * ( 0 ) | 0 *  0 * ( 0 ) |
| S9 mix  (+) | | Negtive control | 102  119 ( 111 ) | 10  11 ( 11 ) | 29  33 ( 31 ) | 26  21 ( 24 ) | 22  23 ( 23 ) |
|  |  | 156 | 121  110 ( 116 ) | 11  13 ( 12 ) | NT | 29  31 ( 30 ) | 24  26 ( 25 ) |
|  |  | 313 | 134  113 ( 124 ) | 5  8 ( 7 ) | 30  28 ( 29 ) | 26  18 ( 22 ) | 22  27 ( 25 ) |
|  |  | 625 | 181  147 ( 164 ) | 12  15 ( 14 ) | 28  33 ( 31 ) | 28  26 ( 27 ) | 27  16 ( 22 ) |
|  |  | 1250 | 268  234 ( 251 ) | 16  17 ( 17 ) | 53  35 ( 44 ) | 29  13 ( 21 ) | 23  15 ( 19 ) |
|  |  | 2500 | 402  354 ( 378 ) | 12  13 ( 13 ) | 46  34 ( 40 ) | 24  23 ( 24 ) | 11  21 ( 16 ) |
|  |  | 5000 | 0 *  0 * ( 0 ) | 7 *  1 * ( 4 ) | 66 *  63 * ( 65 ) | 0 *  0 * ( 0 ) | 0 *  0 * ( 0 ) |
|  | S9 mix  (-) | Positive controls | AF-2 | SA | AF-2 | AF-2 | 9AA |
|  |  | Dose (µg/plate) | 0.01 | 0.5 | 0.01 | 0.1 | 80 |
|  |  | Colonies/plate | 451  434 ( 443 ) | 601  601 ( 601 ) | 136  100 ( 118 ) | 608  579 ( 594 ) | 599  520 ( 560 ) |
|  | S9 mix  (+) | Positive controls | B[a]P | 2AA | 2AA | B[a]P | B[a]P |
|  |  | Dose (µg/plate) | 5 | 2 | 10 | 5 | 5 |
|  |  | Colonies/plate | 1339  1251 ( 1295 ) | 436  467 ( 452 ) | 753  687 ( 720 ) | 478  468 ( 473 ) | 167  176 ( 172 ) |

( ), the average number of colonies. Negative control, DMSO

AF-2, 2-(2-Furyl)-3-(5-nitro-2-furyl)acrylamide; SA, Sodium azide; 9AA, 9-Aminoacridine; B[a]P, Benzo[a]pyrene; 2AA, 2-Aminoanthracene

†, Precipitation

*, Growth inhibition

NT, Not tested

**Appendix Ic: 2-[(methylthio)methyl]-2-butenal (Exp. 3)**

| Metabolic activation | | Dose  (µg/plate) | Colonies /plate | |
| --- | --- | --- | --- | --- |
|  |  |  | Base substitution | Frameshift |
|  |  |  | WP2 *uvrA* | TA98 |
| S9 mix  (-) | | Negative control |  | 17  18 ( 18 ) |
|  |  | 1000 |  | 28  31 ( 30 ) |
|  |  | 2000 |  | 35  34 ( 35 ) |
|  |  | 2500 |  | 29  22 ( 26 ) |
|  |  | 3000 |  | 18  28 ( 23 ) |
|  |  | 4000 |  | 0 *  0 * ( 0 ) |
|  |  | 5000 |  | 0 *  0 * ( 0 ) |
| S9 mix  (+) | | Negative control | 22  26 ( 24 ) |  |
|  |  | 1000 | 39  44 ( 42 ) |  |
|  |  | 2000 | 31  38 ( 35 ) |  |
|  |  | 2500 | 46  40 ( 43 ) |  |
|  |  | 3000 | 47  26 ( 37 ) |  |
|  |  | 4000 | 57  57 ( 57 ) |  |
|  |  | 5000 | 58 *  74 * ( 66 ) |  |
|  | S9 mix  (+) | Positive controls |  | AF-2 |
|  |  | Dose (µg/plate) |  | 0.1 |
|  |  | Colonies/plate |  | 579  492 ( 536 ) |
|  | S9 mix  (+) | Positive controls | 2AA |  |
|  |  | Dose (µg/plate) | 10 |  |
|  |  | Colonies/plate | 650  721 ( 686 ) |  |

( ), the average number of colonies. Negative control, DMSO

AF-2, 2-(2-Furyl)-3-(5-nitro-2-furyl)acrylamide; 2AA, 2-Aminoanthracene

**Appendix Id: 2-[(methylthio)methyl]-2-butenal (Relative Activity Value; RAV)**

|  | Strain | S9 mix (-) | | S9 mix (+) | |
| --- | --- | --- | --- | --- | --- |
|  |  | RAV* | Dose  (μg/plate) | RAV* | Dose  (μg/plate) |
| Exp. 1 | TA100 | 209 | 1500 | 115 | 1500 |
| Exp. 2  Exp. 3 | TA100 | 225 | 1250 | 112 | 1250 |
|  |  | 110 | 2500 | 107 | 2500 |
|  | WP2 *uvrA* | － | － | 7 | 5000 |
| Exp. 3 | WP2 *uvrA* | － | － | 8 | 4000 |
|  |  |  |  | 8 | 5000 |

*: Colonies/mg

**Appendix IIa: 4'-methoxycinnamaldehyde (Exp. 1a)**

| Metabolic  activation | | Dose (µg/plate) | Colonies /plate | | | | |
| --- | --- | --- | --- | --- | --- | --- | --- |
|  |  |  | Base-substitution | | | Frameshift | |
|  |  |  | TA100 | TA1535 | WP2 *uvrA* | TA98 | TA1537 |
| -S9 mix | | Negative control | 133 (128)  123 | 18 ( 14)  9 | 19 ( 21)  23 | 25 ( 23)  20 | 10 ( 10)  10 |
|  |  | 0.305 | 106 (113)  119 | 13 ( 14)  14 | 26 ( 24)  22 | 25 ( 20)  15 | 7 ( 7)  7 |
|  |  | 1.22 | 125 (115)  105 | 16 ( 15)  14 | 24 ( 29)  33 | 20 ( 18)  16 | 8 ( 8)  8 |
|  |  | 4.88 | 115 (120)  124 | 16 ( 13)  9 | 28 ( 28)  28 | 35 ( 28)  20 | 6 ( 8)  10 |
|  |  | 19.5 | 109 (113)  116 | 19 ( 16)  12 | 25 ( 20)  15 | 23 ( 27)  31 | 6 ( 7)  7 |
|  |  | 78.1 | 102 (103)  104 | 15 ( 14)  12 | 21 ( 20)  19 | 21 ( 23)  24 | 8 ( 8)  7 |
|  |  | 313 | 133 (122)  110 | 13 ( 11)  9 | 28 ( 24)  19 | 29 ( 27)  24 | 8 ( 7)  6 |
|  |  | 1250 | 56 * ( 58)  60 * | 8 * ( 8)  8 * | 15 * ( 14)  12 * | 8 * ( 12)  16 * | 4 * ( 5)  5 * |
|  |  | 5000 | 0 * ( 0)  0 * | 0 * ( 0)  0 * | 0 * ( 0)  0 * | 0 * ( 0)  0 * | 0 * ( 0)  0 * |
|  | S9 mix  (-) | Positive controls | AF-2 | AZI | AF-2 | AF-2 | 9AA |
|  |  | Dose (µg/plate) | 0.01 | 0.5 | 0.01 | 0.1 | 80.0 |
|  |  | Colonies/plate | 609 (605)  600 | 638 (625)  612 | 179 (157)  134 | 449 (472)  495 | 234 (241)  248 |

( ), the average number of colonies. Negative control, DMSO

AF-2: 2-(2-Furyl)-3-(5-nitro-2-furyl)acrylamide, AZI: Sodium azide, 9AA: 9-Aminoacridin

*: Growth inhibition

**Appendix IIb: 4'-methoxycinnamaldehyde (Exp. 1b)**

| Metabolic  activation | | Dose (µg/plate) | Colonies /plate | | | | |
| --- | --- | --- | --- | --- | --- | --- | --- |
|  |  |  | Base-substitution | | | Frameshift | |
|  |  |  | TA100 | TA1535 | WP2 *uvrA* | TA100 | TA1537 |
| + S9 mix | | Negative control | 122 (124)  126 | 18 ( 14)  10 | 33 ( 35)  37 | 29 ( 37)  45 | 15 ( 16)  17 |
|  |  | 0.305 | 129 (115)  100 | 12 ( 11)  9 | 33 ( 33)  32 | 33 ( 36)  38 | 15 ( 16)  16 |
|  |  | 1.22 | 120 (119)  118 | 21 ( 19)  16 | 30 ( 31)  32 | 37 ( 33)  28 | 14 ( 14)  14 |
|  |  | 4.88 | 122 (132)  142 | 13 ( 10)  7 | 39 ( 38)  36 | 35 ( 39)  43 | 15 ( 16)  16 |
|  |  | 19.5 | 132 (126)  119 | 12 ( 15)  18 | 33 ( 34)  34 | 39 ( 35)  31 | 12 ( 13)  14 |
|  |  | 78.1 | 165 (154)  142 | 16 ( 15)  13 | 25 ( 30)  35 | 27 ( 29)  31 | 19 ( 19)  18 |
|  |  | 313 | 187 (206)  225 | 16 ( 17)  17 | 27 ( 29)  30 | 34 ( 34)  34 | 14 ( 14)  14 |
|  |  | 1250 | 81 * ( 74)  67 * | 6 * ( 7)  8 * | 25 * ( 23)  21 * | 21 * ( 19)  16 * | 4 * ( 6)  7 * |
|  |  | 5000 | 0 * ( 0)  0 * | 0 * ( 0)  0 * | 0 * ( 0)  0 * | 0 * ( 0)  0 * | 0 * ( 0)  0 * |
|  | S9 mix  (+) | Positive controls | 2AA | 2AA | 2AA | 2AA | 2AA |
|  |  | Dose (µg/plate) | 1.0 | 2.0 | 10.0 | 0.5 | 2.0 |
|  |  | Colonies/plate | 1341 (1350)  1359 | 541 (517)  493 | 1180 (1234)  1288 | 646 (633)  619 | 240 (235)  229 |

( ), the average number of colonies. Negative control, DMSO

2AA: 2-Aminoanthracene

*: Growth inhibition

# **Appendix IIc 4'-methoxycinnamaldehyde (Exp. 1c)**

| Metabolic  activation | | Dose (µg/plate) | Colonies /plate |
| --- | --- | --- | --- |
|  |  |  | Base -substitution |
|  |  |  | TA100 |
| + S9 mix | | Negative control | 121 (126)  130 |
|  |  | 73.2 | 125 (135)  144 |
|  |  | 110 | 161 (157)  152 |
|  |  | 165 | 160 (163)  165 |
|  |  | 247 | 175 (176)  176 |
|  |  | 370 | 252 (206)  160 |
|  |  | 556 | 163 (180)  196 |
|  |  | 833 | 96 * ( 92)  87 * |
|  |  | 1250 | 58 * ( 70)  81 * |
|  | S9 mix  (+) | Positive controls | 2AA |
|  |  | Dose (µg/plate) | 1.0 |
|  |  | Colonies/plate | 1015 (1065)  1114 |

( ), the average number of colonies. Negative control, DMSO

2AA: 2-Aminoanthracene

*: Growth inhibition

# **Appendix IId 4'-methoxycinnamaldehyde (Exp.2)**

| Metabolic  activation | | Dose (µg/plate) | Colonies /plate | | | | |
| --- | --- | --- | --- | --- | --- | --- | --- |
|  |  |  | Base-substitution | | | Frameshift | |
|  |  |  | TA100 | TA1535 | WP2 *uvrA* | TA100 | TA1537 |
| -S9 mix | | Negative control | 102 (107)  111 | 15 ( 18)  20 | 23 ( 23)  23 | 14 ( 15)  15 | 10 ( 11)  11 |
|  |  | 39.1 | 85 ( 94)  103 | 17 ( 17)  17 | 26 ( 27)  27 | 19 ( 18)  16 | 8 ( 8)  8 |
|  |  | 78.1 | 111 (101)  90 | 14 ( 15)  16 | 27 ( 28)  29 | 19 ( 22)  24 | 7 ( 9)  10 |
|  |  | 156 | 104 (107)  109 | 13 ( 14)  14 | 25 ( 28)  31 | 20 ( 18)  16 | 7 ( 8)  9 |
|  |  | 313 | 99 (109)  118 | 8 ( 11)  13 | 24 ( 25)  26 | 12 ( 16)  19 | 12 ( 13)  13 |
|  |  | 625 | 93 ( 92)  91 | 10 ( 13)  16 | 28 ( 24)  20 | 22 ( 21)  19 | 9 * ( 9)  8 * |
|  |  | 1250 | 8 * ( 5)  2 * | 0 * ( 0)  0 * | 17 * ( 17)  16 * | 0 * ( 0)  0 * | 0 * ( 0)  0 * |
| + S9 mix | | Negative control |  | 17 ( 16)  14 | 26 ( 25)  24 | 30 ( 30)  29 | 15 ( 15)  15 |
|  |  | 39.1 |  | 19 ( 19)  19 | 37 ( 32)  27 | 23 ( 22)  21 | 16 ( 15)  13 |
|  |  | 78.1 |  | 12 ( 10)  8 | 20 ( 26)  31 | 33 ( 34)  35 | 15 ( 17)  19 |
|  |  | 156 |  | 11 ( 14)  16 | 31 ( 31)  30 | 26 ( 36)  46 | 18 ( 17)  16 |
|  |  | 313 |  | 12 ( 15)  17 | 28 ( 29)  29 | 29 ( 30)  30 | 15 ( 16)  17 |
|  |  | 625 |  | 15 * ( 16)  16 * | 33 ( 30)  26 | 31 ( 36)  40 | 18 * ( 16)  13 * |
|  |  | 1250 |  | 12 * ( 11)  10 * | 34 * ( 25)  16 * | 16 * ( 17)  17 * | 8 * ( 7)  5 * |
|  | S9 mix  (-) | Positive controls | AF-2 | AZI | AF-2 | AF-2 | 9AA |
|  |  | Dose (µg/plate) | 0.01 | 0.5 | 0.01 | 0.1 | 80.0 |
|  |  | Colonies/plate | 518 (515)  512 | 656 (643)  629 | 164 (144)  123 | 392 (406)  420 | 228 (253)  277 |
|  | S9 mix  (+) | Positive controls | 2AA | 2AA | 2AA | 2AA | 2AA |
|  |  | Dose (µg/plate) | 1.0 | 2.0 | 10.0 | 0.5 | 2.0 |
|  |  | Colonies/plate | 1015 (1065)  1114 | 538 (521)  504 | 1452 (1520)  1587 | 511 (505)  498 | 188 (194)  199 |

( ), the average number of colonies. Negative control, DMSO

AF-2: 2-(2-Furyl)-3-(5-nitro-2-furyl)acrylamide, AZI: Sodium azide, 9AA: 9-Aminoacridine, 2AA: 2-Aminoanthracene

*: Growth inhibition

**Appendix IIIa: 4-methyl-2-pentenal (Exp. 1)**

| Metabolic  activation | | Dose  (µg/plate) | Colonies /plate | | | | |
| --- | --- | --- | --- | --- | --- | --- | --- |
|  |  |  | Base-substitution | | | Frameshift | |
|  |  |  | TA100 | TA1535 | WP2 *uvrA* | TA98 | TA1537 |
| S9 mix  (-) | | Negtive control | 120  101 ( 111 ) | 13  12 ( 13 ) | 42  43 ( 43 ) | 16  21 ( 19 ) | 7  8 ( 8 ) |
|  |  | 1.50 | 119  110 ( 115 ) | 14  18 ( 16 ) | 28  40 ( 34 ) | 14  7 ( 11 ) | 14  14 ( 14 ) |
|  |  | 5.00 | 121  115 ( 118 ) | 17  17 ( 17 ) | 19  36 ( 28 ) | 17  14 ( 16 ) | 9  7 ( 8 ) |
|  |  | 15.0 | 137  102 ( 120 ) | 19  16 ( 18 ) | 39  17 ( 28 ) | 24  12 ( 18 ) | 12  14 ( 13 ) |
|  |  | 50.0 | 161  133 ( 147 ) | 13  24 ( 19 ) | 33  36 ( 35 ) | 19  14 ( 17 ) | 14  7 ( 11 ) |
|  |  | 150 | 310  313 ( 312 ) | 11  18 ( 15 ) | 43  51 ( 47 ) | 18  22 ( 20 ) | 9  13 ( 11 ) |
|  |  | 500 | 2 *  11 * ( 7 ) | 3 *  1 * ( 2 ) | 44 *  19 * ( 32 ) | 0 *  0 * ( 0 ) | 0 *  1 * ( 1 ) |
|  |  | 1500 | 0 *  0 * ( 0 ) | 0 *  0 * ( 0 ) | 0 *  0 * ( 0 ) | 0 *  0 * ( 0 ) | 0 *  0 * ( 0 ) |
|  |  | 5000 | 0 *  0 * ( 0 ) | 0 *  0 * ( 0 ) | 0 *  0 * ( 0 ) | 0 *  0 * ( 0 ) | 0 *  0 * ( 0 ) |
| S9 mix  (+) | | Negative control | 120  146 ( 133 ) | 12  10 ( 11 ) | 41  30 ( 36 ) | 20  39 ( 30 ) | 20  20 ( 20 ) |
|  |  | 1.50 | 102  110 ( 106 ) | 9  13 ( 11 ) | 37  34 ( 36 ) | 12  32 ( 22 ) | 31  17 ( 24 ) |
|  |  | 5.00 | 130  115 ( 123 ) | 12  7 ( 10 ) | 33  41 ( 37 ) | 30  22 ( 26 ) | 24  23 ( 24 ) |
|  |  | 15.0 | 115  123 ( 119 ) | 11  11 ( 11 ) | 33  34 ( 34 ) | 37  25 ( 31 ) | 13  19 ( 16 ) |
|  |  | 50.0 | 142  138 ( 140 ) | 9  10 ( 10 ) | 39  29 ( 34 ) | 17  26 ( 22 ) | 18  15 ( 17 ) |
|  |  | 150 | 230  255 ( 243 ) | 12  8 ( 10 ) | 39  50 ( 45 ) | 24  30 ( 27 ) | 23  16 ( 20 ) |
|  |  | 500 | 83 *  204 * ( 144 ) | 6 *  7 * ( 7 ) | 34  31 ( 33 ) | 24 *  22 * ( 23 ) | 17 *  16 * ( 17 ) |
|  |  | 1500 | 0 *  0 * ( 0 ) | 0 *  0 * ( 0 ) | 0 *  0 * ( 0 ) | 0 *  0 * ( 0 ) | 0 *  0 * ( 0 ) |
|  |  | 5000 † | 0 *  0 * ( 0 ) | 0 *  0 * ( 0 ) | 0 *  0 * ( 0 ) | 0 *  0 * ( 0 ) | 0 *  0 * ( 0 ) |
|  | S9 mix  (-) | Positive controls | AF-2 | SA | AF-2 | AF-2 | 9AA |
|  |  | Dose (µg/plate) | 0.01 | 0.5 | 0.01 | 0.1 | 80 |
|  |  | Colonies/plate | 518  487 ( 503 ) | 611  659 ( 635 ) | 154  164 ( 159 ) | 616  581 ( 599 ) | 481  421 ( 451 ) |
|  | S9 mix  (+) | Positive controls | B[a]P | 2AA | 2AA | B[a]P | B[a]P |
|  |  | Dose (µg/plate) | 5 | 2 | 10 | 5 | 5 |
|  |  | Colonies/plate | 1136  1109 ( 1123 ) | 489  502 ( 496 ) | 709  695 ( 702 ) | 382  370 ( 376 ) | 155  126 ( 141 ) |

( ), the average number of colonies. Negative control, DMSO

AF-2, 2-(2-Furyl)-3-(5-nitro-2-furyl)acrylamide; SA, Sodium azide; 9AA, 9-Aminoacridine; B[a]P, Benzo[a]pyrene; 2AA, 2-Aminoanthracene

†, Precipitation

*, Growth inhibition

| Metabolic  activation | | Dose  (µg/plate) | Colonies /plate | | | | |
| --- | --- | --- | --- | --- | --- | --- | --- |
|  |  |  | Basesubstitution | | | Frameshift | |
|  |  |  | TA100 | TA100 | TA100 | TA100 | TA100 |
| S9 mix  (-) | | Negtive control | 120  127 ( 124 ) | 7  8 ( 8 ) | 29  28 ( 29 ) | 26  19 ( 23 ) | 10  8 ( 9 ) |
|  |  | 15.6 | 127  138 ( 133 ) | 13  6 ( 10 ) | 37  32 ( 35 ) | 21  12 ( 17 ) | 5  8 ( 7 ) |
|  |  | 31.3 | 134  127 ( 131 ) | 7  10 ( 9 ) | 32  35 ( 34 ) | 18  14 ( 16 ) | 6  6 ( 6 ) |
|  |  | 62.5 | 158  185 ( 172 ) | 11  4 ( 8 ) | 37  23 ( 30 ) | 11  15 ( 13 ) | 13  10 ( 12 ) |
|  |  | 125 | 247  235 ( 241 ) | 11  8 ( 10 ) | 40  32 ( 36 ) | 18  19 ( 19 ) | 12  13 ( 13 ) |
|  |  | 250 | 410  325 ( 368 ) | 10  8 ( 9 ) | 28  34 ( 31 ) | 17  31 ( 24 ) | 6  13 ( 10 ) |
|  |  | 500 | 1 *  9 * ( 5 ) | 1 *  2 * ( 2 ) | 21 *  30 * ( 26 ) | 1 *  3 * ( 2 ) | 0 *  0 * ( 0 ) |
|  |  | 1000 | 0 *  0 * ( 0 ) | 0 *  0 * ( 0 ) | 0 *  0 * ( 0 ) | 0 *  0 * ( 0 ) | 0 *  0 * ( 0 ) |
| S9 mix  (+) | | Negtive control | 128  123 ( 126 ) | 7  11 ( 9 ) | 26  28 ( 27 ) | 30  26 ( 28 ) | 11  22 ( 17 ) |
|  |  | 15.6 | 134  144 ( 139 ) | 16  12 ( 14 ) | NT | 22  27 ( 25 ) | 18  13 ( 16 ) |
|  |  | 31.3 | 133  143 ( 138 ) | 10  7 ( 9 ) | NT | 31  24 ( 28 ) | 18  21 ( 20 ) |
|  |  | 62.5 | 174  152 ( 163 ) | 12  4 ( 8 ) | 27  34 ( 31 ) | 22  34 ( 28 ) | 24  20 ( 22 ) |
|  |  | 125 | 188  181 ( 185 ) | 13  13 ( 13 ) | 25  44 ( 35 ) | 27  37 ( 32 ) | 21  16 ( 19 ) |
|  |  | 250 | 317  299 ( 308 ) | 16  13 ( 15 ) | 27  32 ( 30 ) | 34  26 ( 30 ) | 28  16 ( 22 ) |
|  |  | 500 | 205 *  198 * ( 202 ) | 6 *  6 * ( 6 ) | 32  34 ( 33 ) | 22 *  20 * ( 21 ) | 6 *  13 * ( 10 ) |
|  |  | 1000 | 0 *  0 * ( 0 ) | 0 *  0 * ( 0 ) | 0 *  0 * ( 0 ) | 0 *  0 * ( 0 ) | 0 *  0 * ( 0 ) |
|  |  | 2000 | NT | NT | 0 *  0 * ( 0 ) | NT | NT |
|  | S9 mix  (-) | Potitive controls | AF-2 | SA | AF-2 | AF-2 | 9AA |
|  |  | Dose (µg/plate) | 0.01 | 0.5 | 0.01 | 0.1 | 80 |
|  |  | Colonies/plate | 457  418 ( 438 ) | 666  689 ( 678 ) | 141  117 ( 129 ) | 547  578 ( 563 ) | 418  490 ( 454 ) |
|  | S9 mix  (+) | Potitive controls | B[a]P | 2AA | 2AA | B[a]P | B[a]P |
|  |  | Dose (µg/plate) | 5 | 2 | 10 | 5 | 5 |
|  |  | Colonies/plate | 1323  1208 ( 1266 ) | 514  477 ( 496 ) | 624  652 ( 638 ) | 461  487 ( 474 ) | 194  178 ( 186 ) |

**Appendix IIIb: 4-methyl-2-pentenal (Exp. 2)**

( ), the average number of colonies. Negative control, DMSO

AF-2, 2-(2-Furyl)-3-(5-nitro-2-furyl)acrylamide; SA, Sodium azide; 9AA, 9-Aminoacridine; B[a]P, Benzo[a]pyrene; 2AA, 2-Aminoanthracene

*: Growth inhibition

NT, Not tested

**Appendix IIIc: 4-methyl-2-pentenal (Exp. 3)**

| Metabolic activation | | Dose  (µg/plate) | Colonies /plate | |
| --- | --- | --- | --- | --- |
|  |  |  | Base substitution | |
|  |  |  | TA100 | |
|  | | Negative | 130 |  |
|  | | control | 132 | ( 131 ) |
|  | | 100 | 138 |  |
|  | |  | 106 | ( 122 ) |
|  | | 150 | 133 |  |
|  | |  | 118 | ( 126 ) |
| S9 mix | | 200 | 127 |  |
|  | |  | 123 | ( 125 ) |
|  | | 250 | 184 |  |
| (+) | |  | 178 | ( 181 ) |
|  | | 300 | 290 |  |
|  | |  | 314 | ( 302 ) |
|  | | 400 | 394 |  |
|  | |  | 375 | ( 385 ) |
|  | | 500 | 125 * |  |
|  | |  | 162 * | ( 144 ) |
|  | S9 mix  (+) | Positive controls | B[a]P | |
|  |  | Dose (µg/plate) | 5 | |
|  |  | Colonies/plate | 1430  1282 | ( 1356 ) |

( ), the average number of colonies. Negative control, DMSO

B[a]P, Benzo[a]pyrene

*, Growth inhibition

**Appendix IIId: 4-methyl-2-pentenal (Relative Activity Value; RAV)**

|  | Strain | S9 mix (-) | | S9 mix (+) | |
| --- | --- | --- | --- | --- | --- |
|  |  | RAV* | Dose  (μg/plate) | RAV* | Dose  (μg/plate) |
| Exp. 1 | TA100 | 1340 | 150 | － | － |
| Exp. 2 | TA100 | 976 | 250 | 728 | 250 |
| Exp. 3 | TA100 | － | － | 570 | 300 |
|  |  |  |  | 635 | 400 |

*: Colonies/m

**Appendix IVa: 4-methoxy-2,5-dimethyl-3(2H)-furanone (Exp. 1)**

| Metabolic  activation | | Dose  (µg/plate) | Colonies /plate | | | | |
| --- | --- | --- | --- | --- | --- | --- | --- |
|  |  |  | Base-substitution | | | Frameshift | |
|  |  |  | TA100 | TA1535 | WP2 *uvrA* | TA98 | TA1537 |
| -S9 mix | | Negative control | 138 (121)  104 | 12 ( 13)  13 | 27 ( 26)  24 | 22 ( 25)  27 | 7 ( 7)  7 |
|  |  | 0.305 | 136 (136)  136 | 9 ( 14)  18 | 18 ( 19)  19 | 31 ( 30)  29 | 3 ( 4)  5 |
|  |  | 1.22 | 119 (115)  110 | 15 ( 14)  12 | 19 ( 20)  21 | 31 ( 32)  32 | 4 ( 6)  8 |
|  |  | 4.88 | 114 (112)  109 | 16 ( 14)  12 | 28 ( 24)  19 | 29 ( 29)  29 | 7 ( 8)  8 |
|  |  | 19.5 | 119 (114)  108 | 9 ( 9)  8 | 19 ( 20)  21 | 24 ( 22)  19 | 7 ( 8)  9 |
|  |  | 78.1 | 121 (124)  126 | 13 ( 13)  13 | 24 ( 24)  23 | 23 ( 25)  26 | 6 ( 7)  8 |
|  |  | 313 | 133 (122)  110 | 10 ( 13)  15 | 22 ( 24)  26 | 29 ( 27)  24 | 7 ( 6)  4 |
|  |  | 1250 | 112 (108)  104 | 14 ( 17)  19 | 31 ( 31)  30 | 29 ( 29)  29 | 4 ( 6)  7 |
|  |  | 5000 | 118 (131)  144 | 11 ( 12)  12 | 26 ( 27)  27 | 17 ( 25)  33 | 5 ( 7)  8 |
|  | S9 mix  (-) | Positive controls | AF-2 | AZI | AF-2 | AF-2 | 9AA |
|  |  | Dose (µg/plate) | 0.01 | 0.5 | 0.01 | 0.1 | 80.0 |
|  |  | Colonies/plate | 609 (605)  600 | 638 (625)  612 | 179 (157)  134 | 449 (472)  495 | 234 (241)  248 |

( ), the average number of colonies. Negative control, Saline

AF-2: 2-(2-Furyl)-3-(5-nitro-2-furyl)acrylamide, AZI: Sodium azide, 9AA: 9-Aminoacridine

**Appendix IVb: 4-methoxy-2,5-dimethyl-3(2H)-furanone (Exp. 1b)**

| Metabolic  activation | | Dose  (µg/plate) | Colonies /plate | | | | |
| --- | --- | --- | --- | --- | --- | --- | --- |
|  |  |  | Base-substitution | | | Frameshift | |
|  |  |  | TA100 | TA1535 | WP2 *uvrA* | TA98 | TA1537 |
| + S9 mix | | Negative control | 133 (128)  123 | 8 ( 14)  19 | 20 ( 23)  25 | 22 ( 28)  34 | 16 ( 16)  15 |
|  |  | 0.305 | 127 (135)  142 | 16 ( 14)  12 | 26 ( 27)  28 | 31 ( 34)  37 | 19 ( 17)  15 |
|  |  | 1.22 | 116 (125)  134 | 12 ( 10)  8 | 24 ( 23)  21 | 45 ( 43)  40 | 14 ( 14)  14 |
|  |  | 4.88 | 121 (121)  120 | 9 ( 10)  11 | 34 ( 30)  25 | 32 ( 29)  25 | 15 ( 16)  16 |
|  |  | 19.5 | 123 (119)  114 | 14 ( 11)  7 | 27 ( 29)  31 | 29 ( 29)  29 | 15 ( 15)  15 |
|  |  | 78.1 | 135 (126)  116 | 10 ( 11)  11 | 30 ( 29)  27 | 26 ( 28)  29 | 12 ( 14)  16 |
|  |  | 313 | 121 (125)  129 | 18 ( 16)  14 | 27 ( 29)  30 | 32 ( 39)  45 | 17 ( 16)  15 |
|  |  | 1250 | 122 (127)  131 | 15 ( 14)  12 | 31 ( 28)  24 | 31 ( 29)  26 | 14 ( 15)  15 |
|  |  | 5000 | 119 (119)  119 | 13 ( 13)  12 | 31 ( 30)  29 | 41 ( 34)  26 | 13 ( 11)  8 |
|  | S9 mix  (+) | Positive controls | 2AA | 2AA | 2AA | 2AA | 2AA |
|  |  | Dose (µg/plate) | 1.0 | 2.0 | 10.0 | 0.5 | 2.0 |
|  |  | Colonies/plate | 1341 (1350)  1359 | 541 (517)  493 | 1180 (1234)  1288 | 646 (633)  619 | 240 (235)  229 |

( ), the average number of colonies. Negative control, Saline

2AA: 2-Aminoanthracene

**Appendix IVc: 4-methoxy-2,5-dimethyl-3(2H)-furanone (Exp. 2)**

| Metabolic  activation | | Dose  (µg/plate) | Colonies/plate | | | | |
| --- | --- | --- | --- | --- | --- | --- | --- |
|  |  |  | Base-substitution | | | Frameshift | |
|  |  |  | TA100 | TA1535 | WP2 *uvrA* | TA98 | TA1537 |
| -S9 mix | | Negative control | 103 (107)  110 | 11 ( 10)  8 | 23 ( 26)  28 | 14 ( 16)  17 | 8 ( 7)  6 |
|  |  | 313 | 96 (104)  112 | 17 ( 14)  10 | 26 ( 23)  19 | 17 ( 16)  14 | 13 ( 12)  11 |
|  |  | 625 | 125 (119)  112 | 14 ( 12)  10 | 17 ( 22)  27 | 20 ( 20)  19 | 11 ( 10)  9 |
|  |  | 1250 | 88 (106)  123 | 12 ( 15)  18 | 23 ( 22)  20 | 17 ( 15)  13 | 8 ( 8)  7 |
|  |  | 2500 | 110 (111)  111 | 11 ( 12)  12 | 28 ( 25)  21 | 26 ( 25)  24 | 7 ( 9)  10 |
|  |  | 5000 | 102 (102)  102 | 12 ( 15)  18 | 21 ( 22)  23 | 21 ( 18)  14 | 9 ( 11)  12 |
| + S9 mix | | Negative control | 111 (116)  120 | 13 ( 16)  18 | 24 ( 22)  19 | 35 ( 27)  18 | 17 ( 16)  15 |
|  |  | 313 | 114 (120)  126 | 17 ( 19)  20 | 28 ( 34)  39 | 27 ( 27)  27 | 16 ( 17)  17 |
|  |  | 625 | 109 (104)  99 | 13 ( 14)  14 | 22 ( 23)  23 | 26 ( 26)  26 | 19 ( 17)  15 |
|  |  | 1250 | 118 (106)  93 | 16 ( 13)  9 | 24 ( 23)  21 | 22 ( 27)  31 | 19 ( 18)  17 |
|  |  | 2500 | 92 ( 96)  100 | 12 ( 14)  15 | 29 ( 29)  29 | 31 ( 27)  23 | 18 ( 19)  19 |
|  |  | 5000 | 91 ( 93)  94 | 9 ( 11)  13 | 33 ( 32)  30 | 23 ( 22)  21 | 12 ( 14)  15 |
|  | S9 mix  (-) | Positive controls | AF-2 | AZI | AF-2 | AF-2 | 9AA |
|  |  | Dose (µg/plate) | 0.01 | 0.5 | 0.01 | 0.1 | 80.0 |
|  |  | Colonies/plate | 518 (515)  512 | 656 (643)  629 | 164 (144)  123 | 392 (406)  420 | 228 (253)  277 |
|  | S9 mix  (+) | Positive controls | 2AA | 2AA | 2AA | 2AA | 2AA |
|  |  | Dose (µg/plate) | 1.0 | 2.0 | 10.0 | 0.5 | 2.0 |
|  |  | Colonies/plate | 1015 (1065)  1114 | 538 (521)  504 | 1452 (1520)  1587 | 511 (505)  498 | 188 (194)  199 |

( ), the average number of colonies. Negative control, Saline

AF-2: 2-(2-Furyl)-3-(5-nitro-2-furyl)acrylamide, AZI: Sodium azide, 9AA: 9-Aminoacridine, 2AA: 2-Aminoanthracene

## Appendix Va: 2,5-dimethyl-4-oxo-3(5H)-furyl acetate (Exp. 1)

| Metabolic  activation | | Dose  (µg/plate) | Colonies /plate | | | | |
| --- | --- | --- | --- | --- | --- | --- | --- |
|  |  |  | Base substitution | | | Frameshift | |
|  |  |  | TA100 | TA1535 | WP2 *uvrA* | TA98 | TA1537 |
| S9 mix  (-) | | Negtive control | 136  131 ( 134 ) | 6  17 ( 12 ) | 37  25 ( 31 ) | 17  20 ( 19 ) | 12  14 ( 13 ) |
|  |  | 50.0 | 130  122 ( 126 ) | 8  16 ( 12 ) | 42  36 ( 39 ) | 12  20 ( 16 ) | 10  7 ( 9 ) |
|  |  | 150 | 152  151 ( 152 ) | 9  11 ( 10 ) | 37  32 ( 35 ) | 24  23 ( 24 ) | 9  12 ( 11 ) |
|  |  | 500 | 179  161 ( 170 ) | 14  11 ( 13 ) | 35  32 ( 34 ) | 21  20 ( 21 ) | 7  16 ( 12 ) |
|  |  | 1500 | 208  242 ( 225 ) | 8  12 ( 10 ) | 31  33 ( 32 ) | 11  27 ( 19 ) | 11  4 ( 8 ) |
|  |  | 5000 | 325  351 ( 338 ) | 10  10 ( 10 ) | 39  42 ( 41 ) | 23  29 ( 26 ) | 11  9 ( 10 ) |
| S9 mix  (+) | | 0  (Negtive control) | 145  163 ( 154 ) | 11  10 ( 11 ) | 40  40 ( 40 ) | 20  35 ( 28 ) | 13  17 ( 15 ) |
|  |  | 50.0 | 169  152 ( 161 ) | 18  13 ( 16 ) | 36  33 ( 35 ) | 35  24 ( 30 ) | 14  18 ( 16 ) |
|  |  | 150 | 191  149 ( 170 ) | 18  18 ( 18 ) | 32  41 ( 37 ) | 21  27 ( 24 ) | 17  23 ( 20 ) |
|  |  | 500 | 232  197 ( 215 ) | 12  15 ( 14 ) | 30  41 ( 36 ) | 32  36 ( 34 ) | 16  19 ( 18 ) |
|  |  | 1500 | 261  263 ( 262 ) | 17  18 ( 18 ) | 40  30 ( 35 ) | 29  29 ( 29 ) | 18  14 ( 16 ) |
|  |  | 5000 | 383  443 ( 413 ) | 15  14 ( 15 ) | 58  49 ( 54 ) | 37  33 ( 35 ) | 17  23 ( 20 ) |
|  | S9 mix  (-) | Positive controls | AF-2 | SA | AF-2 | AF-2 | 9AA |
|  |  | Dose (µg/plate) | 0.01 | 0.5 | 0.01 | 0.1 | 80 |
|  |  | Colonies/plate | 500  483 ( 492 ) | 645  606 ( 626 ) | 182  144 ( 163 ) | 598  557 ( 578 ) | 428  469 ( 449 ) |
|  | S9 mix  (+) | Positive controls | B[a]P | 2AA | 2AA | B[a]P | B[a]P |
|  |  | Dose (µg/plate) | 5 | 2 | 10 | 5 | 5 |
|  |  | Colonies/plate | 1073  1160 ( 1117 ) | 446  490 ( 468 ) | 631  597 ( 614 ) | 393  356 ( 375 ) | 140  141 ( 141 ) |

( ), the average number of colonies. Negative control, Saline

AF-2, 2-(2-Furyl)-3-(5-nitro-2-furyl)acrylamide; SA, Sodium azide; 9AA, 9-Aminoacridine; B[a]P, Benzo[a]pyrene; 2AA, 2-Aminoanthracene

## Appendix Vb: 2,5-dimethyl-4-oxo-3(5H)-furyl acetate (Exp. 2)

| Metabolic  activation | | Dose  (µg/plate) | Colonies /plate | | | | |
| --- | --- | --- | --- | --- | --- | --- | --- |
|  |  |  | Base substitution | | | Flameshift | |
|  |  |  | TA100 | TA100 | TA100 | TA100 | TA100 |
| S9 mix  (-) | | 0  (Negtive control) | 111  117 ( 114 ) | 10  16 ( 13 ) | 24  35 ( 30 ) | 20  11 ( 16 ) | 7  17 ( 12 ) |
|  |  | 313 | 138  141 ( 140 ) | 13  12 ( 13 ) | 26  28 ( 27 ) | 17  17 ( 17 ) | 7  7 ( 7 ) |
|  |  | 625 | 158  133 ( 146 ) | 15  13 ( 14 ) | 22  25 ( 24 ) | 16  20 ( 18 ) | 5  5 ( 5 ) |
|  |  | 1250 | 176  186 ( 181 ) | 10  16 ( 13 ) | 39  22 ( 31 ) | 12  20 ( 16 ) | 15  4 ( 10 ) |
|  |  | 2500 | 246  242 ( 244 ) | 13  19 ( 16 ) | 25  31 ( 28 ) | 21  18 ( 20 ) | 6  10 ( 8 ) |
|  |  | 5000 | 318  299 ( 309 ) | 12  13 ( 13 ) | 41  30 ( 36 ) | 16  23 ( 20 ) | 7  7 ( 7 ) |
| S9 mix  (+) | | 0  (Negtive control) | 122  156 ( 139 ) | 8  13 ( 11 ) | 30  33 ( 32 ) | 30  28 ( 29 ) | 15  20 ( 18 ) |
|  |  | 313 | 193  177 ( 185 ) | 8  13 ( 11 ) | 30  28 ( 29 ) | 25  27 ( 26 ) | 14  25 ( 20 ) |
|  |  | 625 | 176  172 ( 174 ) | 14  14 ( 14 ) | 36  34 ( 35 ) | 33  29 ( 31 ) | 20  15 ( 18 ) |
|  |  | 1250 | 251  253 ( 252 ) | 12  13 ( 13 ) | 28  30 ( 29 ) | 30  30 ( 30 ) | 25  19 ( 22 ) |
|  |  | 2500 | 350  313 ( 332 ) | 13  13 ( 13 ) | 43  36 ( 40 ) | 28  32 ( 30 ) | 19  26 ( 23 ) |
|  |  | 5000 | 407  397 ( 402 ) | 18  17 ( 18 ) | 47  54 ( 51 ) | 38  40 ( 39 ) | 21  10 ( 16 ) |
|  | S9 mix  (-) | Potitive controls | AF-2 | SA | AF-2 | AF-2 | 9AA |
|  |  | Dose (µg/plate) | 0.01 | 0.5 | 0.01 | 0.1 | 80 |
|  |  | Colonies/plate | 520  514 ( 517 ) | 618  641 ( 630 ) | 149  171 ( 160 ) | 624  619 ( 622 ) | 582  493 ( 538 ) |
|  | S9 mix  (+) | Potitive controls | B[a]P | 2AA | 2AA | B[a]P | B[a]P |
|  |  | Dose (µg/plate) | 5 | 2 | 10 | 5 | 5 |
|  |  | Colonies/plate | 1276  1172 ( 1224 ) | 482  497 ( 490 ) | 726  784 ( 755 ) | 368  367 ( 368 ) | 162  161 ( 162 ) |

( ), the average number of colonies. Negative control, Saline

AF-2, 2-(2-Furyl)-3-(5-nitro-2-furyl)acrylamide; SA, Sodium azide; 9AA, 9-Aminoacridine; B[a]P, Benzo[a]pyrene; 2AA, 2-Aminoanthracene

**Appendix Vc: 2,5-dimethyl-4-oxo-3(5H)-furyl acetate (Relative Activity Value; RAV)**

|  | Strain | S9 mix (-) | | S9 mix (+) | |
| --- | --- | --- | --- | --- | --- |
|  |  | RAV* | Dose  (μg/plate) | RAV* | Dose  (μg/plate) |
| Exp. 1 | TA100 | 41 | 5000 | 52 | 5000 |
| Exp. 2 | TA100 | 52 | 2500 | 77 | 2500 |
|  |  | 39 | 5000 | 53 | 5000 |

*: Colonies/m

## Appendix VIa: 3-acetyl-2,5-dimethylfuran (Exp. 1a)

| Metabolic  activation | | Dose  (µg/plate) | Colonies /plate | | | | |
| --- | --- | --- | --- | --- | --- | --- | --- |
|  |  |  | Base substitution | | | Frameshift | |
|  |  |  | TA100 | TA1535 | WP2 *uvrA* | TA100 | TA1537 |
| -S9 mix | | Negative control | 104 (105)  106 | 16 ( 13)  10 | 25 ( 21)  17 | 13 ( 14)  14 | 14 ( 13)  12 |
|  |  | 0.305 | 111 (123)  135 | 11 ( 13)  15 | 18 ( 22)  26 | 17 ( 17)  17 | 9 ( 11)  12 |
|  |  | 1.22 | 123 (120)  117 | 16 ( 14)  12 | 24 ( 21)  18 | 16 ( 15)  14 | 10 ( 11)  12 |
|  |  | 4.88 | 128 (124)  119 | 10 ( 14)  18 | 19 ( 22)  25 | 13 ( 15)  17 | 13 ( 14)  15 |
|  |  | 19.5 | 116 (125)  133 | 12 ( 14)  16 | 22 ( 22)  21 | 15 ( 15)  15 | 13 ( 12)  11 |
|  |  | 78.1 | 134 (129)  124 | 12 ( 15)  17 | 21 ( 25)  29 | 21 ( 18)  15 | 9 ( 10)  11 |
|  |  | 313 | 128 (136)  143 | 9 ( 13)  16 | 18 ( 20)  22 | 19 ( 19)  19 | 9 ( 10)  10 |
|  |  | 1250 | 982 (938)  894 | 12 ( 14)  15 | 21 ( 23)  24 | 35 ( 41)  46 | 8 ( 9)  9 |
|  |  | 5000 | 0 * ( 0)  0 * | 0 * ( 0)  0 * | 0 * ( 0)  0 * | 0 * ( 0)  0 * | 0 * ( 0)  0 * |
|  | S9 mix  (-) | Positive controls | AF-2 | AZI | AF-2 | AF-2 | 9AA |
|  |  | Dose (µg/plate) | 0.01 | 0.5 | 0.01 | 0.1 | 80.0 |
|  |  | Colonies/plate | 609 (605)  600 | 638 (625)  612 | 179 (157)  134 | 449 (472)  495 | 234 (241)  248 |

( ), the average number of colonies. Negative control, DMSO

AF-2: 2-(2-Furyl)-3-(5-nitro-2-furyl)acrylamide, AZI: Sodium azide, 9AA: 9-Aminoacridine

*, Growth inhibition

## Appendix VIb: 3-acetyl-2,5-dimethylfuran (Exp. 1b)

| Metabolic  activation | | Dose  (µg/plate) | Colonies /plate | | | | |
| --- | --- | --- | --- | --- | --- | --- | --- |
|  |  |  | Base substitution | | | Frameshift | |
|  |  |  | TA100 | TA1535 | WP2 *uvrA* | TA100 | TA1537 |
| + S9 mix | | Negative control | 134 (131)  127 | 7 ( 12)  16 | 32 ( 29)  26 | 31 ( 33)  34 | 18 ( 17)  16 |
|  |  | 0.305 | 129 (132)  135 | 13 ( 11)  8 | 21 ( 21)  20 | 26 ( 27)  28 | 18 ( 17)  16 |
|  |  | 1.22 | 133 (125)  116 | 14 ( 14)  14 | 37 ( 32)  27 | 30 ( 28)  26 | 17 ( 17)  16 |
|  |  | 4.88 | 143 (137)  130 | 14 ( 12)  10 | 25 ( 25)  24 | 23 ( 29)  34 | 15 ( 16)  17 |
|  |  | 19.5 | 122 (126)  130 | 15 ( 12)  9 | 24 ( 25)  26 | 42 ( 31)  20 | 15 ( 16)  16 |
|  |  | 78.1 | 128 (133)  138 | 11 ( 13)  15 | 20 ( 22)  23 | 21 ( 24)  27 | 18 ( 19)  19 |
|  |  | 313 | 123 (129)  134 | 13 ( 11)  8 | 17 ( 23)  28 | 29 ( 25)  20 | 16 ( 17)  17 |
|  |  | 1250 | 156 (149)  141 | 9 ( 10)  11 | 22 ( 22)  22 | 22 ( 24)  26 | 14 ( 15)  15 |
|  |  | 5000 | 0 * ( 0)  0 * | 0 * ( 0)  0 * | 0 * ( 0)  0 * | 0 * ( 0)  0 * | 0 * ( 0)  0 * |
|  | S9 mix  (-) | Positive controls | 2AA | 2AA | 2AA | 2AA | 2AA |
|  |  | Dose (µg/plate) | 1.0 | 2.0 | 10.0 | 0.5 | 2.0 |
|  |  | Colonies/plate | 1341 (1350)  1359 | 541 (517)  493 | 1180 (1234)  1288 | 646 (633)  619 | 240 (235)  229 |

( ), the average number of colonies. Negative control, DMSO

2AA: 2-Aminoanthracene

*, Growth inhibition

## Appendix VIc: 3-acetyl-2,5-dimethylfuran (Exp. 2a)

| Metabolic  activation | | Dose  (µg/plate) | Colonies/plate | |
| --- | --- | --- | --- | --- |
|  |  |  | Base substitution | Frameshift |
|  |  |  | TA100 | TA98 |
| - S9 mix | | Negative control | 145 (124)  103 | 20 ( 22)  24 |
|  |  | 329 | 175 (193)  210 | 21 ( 21)  20 |
|  |  | 494 | 385 (384)  382 | 24 ( 27)  29 |
|  |  | 741 | 1002 (1073)  1144 | 48 ( 46)  43 |
|  |  | 1111 | 1457 (1342)  1226 | 86 ( 73)  59 |
|  |  | 1667 | 1277 (1236)  1195 | 55 ( 74)  93 |
|  |  | 2500 | 78 * ( 97)  115 * | 11 * ( 10)  9 * |
|  | S9 mix  (-) | Positive controls | AF-2 | AF-2 |
|  |  | Dose (µg/plate) | 0.01 | 0.1 |
|  |  | Colonies/plate | 518 (515)  512 | 392 (406)  420 |

( ), the average number of colonies. Negative control, DMSO

AF-2: 2-(2-Furyl)-3-(5-nitro-2-furyl)acrylamide,

*, Growth inhibition

## Appendix VId: 3-acetyl-2,5-dimethylfuran (Exp. 2b)

| Metabolic  activation | | Dose  (µg/plate) | Colonies /plate | | | | |
| --- | --- | --- | --- | --- | --- | --- | --- |
|  |  |  | Base substitution | | | Frameshift | |
|  |  |  | TA100 | TA1535 | WP2 *uvrA* | TA100 | TA1537 |
| -S9 mix | | Negative control |  | 13 ( 12)  11 | 19 ( 24)  28 |  | 9 ( 10)  10 |
|  |  | 156 |  | 12 ( 12)  11 | 30 ( 28)  25 |  | 11 ( 10)  9 |
|  |  | 313 |  | 15 ( 14)  13 | 18 ( 18)  18 |  | 9 ( 10)  10 |
|  |  | 625 |  | 12 ( 13)  14 | 14 ( 18)  22 |  | 11 ( 10)  8 |
|  |  | 1250 |  | 15 ( 16)  17 | 69 ( 59)  49 |  | 9 ( 11)  13 |
|  |  | 2500 |  | 5 * ( 5)  5 * | 27 * ( 23)  18 * |  | 3 * ( 2)  0 * |
|  |  | 5000 |  | 5 * ( 6)  6 * | 16 * ( 18)  19 * |  | 3 * ( 3)  3 * |
| + S9 mix | | Negative control | 143 (136)  129 | 16 ( 16)  15 | 36 ( 31)  25 | 30 ( 31)  31 | 17 ( 16)  15 |
|  |  | 156 | 112 (120)  127 | 14 ( 13)  11 | 20 ( 25)  29 | 39 ( 33)  27 | 17 ( 16)  15 |
|  |  | 313 | 122 (125)  128 | 15 ( 15)  14 | 29 ( 30)  30 | 31 ( 30)  29 | 13 ( 14)  14 |
|  |  | 625 | 135 (128)  121 | 12 ( 11)  10 | 19 ( 20)  21 | 35 ( 33)  30 | 13 ( 13)  13 |
|  |  | 1250 | 251 (245)  239 | 12 ( 14)  16 | 20 ( 23)  26 | 26 ( 30)  34 | 16 ( 15)  14 |
|  |  | 2500 | 273 * (327)  381 * | 13 * ( 10)  6 * | 24 * ( 20)  15 * | 19 * ( 23)  27 * | 8 * ( 5)  2 * |
|  |  | 5000 | 0 * ( 0)  0 * | 0 * ( 0)  0 * | 13 * ( 11)  8 * | 0 * ( 0)  0 * | 0 * ( 0)  0 * |
|  | S9 mix  (-) | Positive controls | AF-2 | AZI | AF-2 | AF-2 | 9AA |
|  |  | Dose (µg/plate) | 0.01 | 0.5 | 0.01 | 0.1 | 80.0 |
|  |  | Colonies/plate | 518 (515)  512 | 656 (643)  629 | 164 (144)  123 | 392 (406)  420 | 228 (253)  277 |
|  | S9 mix  (+) | Positive controls | 2AA | 2AA | 2AA | 2AA | 2AA |
|  |  | Dose (µg/plate) | 1.0 | 2.0 | 10.0 | 0.5 | 2.0 |
|  |  | Colonies/plate | 1015 (1065)  1114 | 538 (521)  504 | 1452 (1520)  1587 | 511 (505)  498 | 188 (194)  199 |

( ), the average number of colonies. Negative control, DMSO

AF-2: 2-(2-Furyl)-3-(5-nitro-2-furyl)acrylamide, AZI: Sodium azide, 9AA: 9-Aminoacridine, 2AA: 2-Aminoanthracene

*, Growth inhibition

## Appendix VIe: 3-acetyl-2,5-dimethylfuran (Exp. 3)

| Metabolic  activation | | Dose  (µg/plate) | Colonies/plate | |
| --- | --- | --- | --- | --- |
|  |  |  | Base substitution | |
|  |  |  | TA100 | WP2 *uvrA* |
| - S9 mix | | Negative control | 124 (126)  127 | 29 ( 28)  27 |
|  |  | 329 |  | 25 ( 25)  25 |
|  |  | 494 |  | 33 ( 38)  42 |
|  |  | 741 |  | 21 ( 45)  68 |
|  |  | 1111 |  | 52 ( 53)  54 |
|  |  | 1667 |  | 163 * (157)  150 * |
|  |  | 2500 |  | 16 * ( 18)  19 * |
| + S9 mix | | Negative control | 122 (128)  134 |  |
|  |  | 329 | 159 (140)  120 |  |
|  |  | 494 | 138 (133)  128 |  |
|  |  | 741 | 203 (182)  160 |  |
|  |  | 1111 | 273 (292)  310 |  |
|  |  | 1667 | 510 * (499)  488 * |  |
|  |  | 2500 | 179 * (131)  82 * |  |
|  | S9 mix  (-) | Positive controls | AF-2 | AF-2 |
|  |  | Dose (µg/plate) | 0.01 | 0.01 |
|  |  | Colonies/plate | 697 (680)  663 | 151 (143)  135 |
|  | S9 mix  (+) | Positive controls | 2AA |  |
|  |  | Dose (µg/plate) | 1.0 |  |
|  |  | Colonies/plate | 1432 (1459)  1485 |  |

( ), the average number of colonies. Negative control, DMSO

AF-2: 2-(2-Furyl)-3-(5-nitro-2-furyl)acrylamide, 2AA: 2-Aminoanthracene

*, Growth inhibition

**Appendix VId:** **3-acetyl-2,5-dimethylfuran (Relative Activity Value; RAV)**

|  | Strain | S9 mix (-) | | S9 mix (+) | |
| --- | --- | --- | --- | --- | --- |
|  |  | RAV* | Dose  (μg/plate) | RAV* | Dose  (μg/plate) |
| Exp. 1  Exp. 2  Exp. 3 | TA100 | 666 | 1250 µg/plate |  |  |
|  | TA1535 |  |  |  |  |
|  | WP2 *uvrA* |  |  |  |  |
|  | TA98 | 21.6 | 1250 µg/plate |  |  |
|  | TA1537 |  |  |  |  |
| Exp. 2  本試験  Exp. 1  Exp. 2 | TA100 | 1281 | 741 µg/plate | 76.4 | 2500 µg/plate |
|  | TA1535 |  |  |  |  |
|  | WP2 *uvrA* | 28.0 | 1250 µg/plate |  |  |
|  | TA98 | 45.9 | 1111 µg/plate |  |  |
|  | TA1537 |  |  |  |  |
| Exp. 3 | TA100 | ――― | ――― | 223 | 1667 µg/plate |
|  | WP2 *uvrA* | 77.4 | 1667 µg/plate | ――― | ――― |
|  |  |  |  |  |  |

*: Colonies/mg

**Appendix VIIa:** **furfuryl formate (Exp. 1)**

| Metabolic activation | | Dose  (µg/plate) | Colonies/plate | | | | |
| --- | --- | --- | --- | --- | --- | --- | --- |
|  |  |  | Base substitution | | | Frameshift | |
|  |  |  | TA100 | TA1535 | WP2 *uvrA* | TA98 | TA1537 |
| S9 mix  (-) | | Negative control | 119  120 ( 120 ) | 7  20 ( 14 ) | 20  20 ( 20 ) | 17  16 ( 17 ) | 10  16 ( 13 ) |
|  |  | 15.0 | 109  90 ( 100 ) | 11  9 ( 10 ) | 29  10 ( 20 ) | 29  18 ( 24 ) | 10  9 ( 10 ) |
|  |  | 50.0 | 136  130 ( 133 ) | 12  8 ( 10 ) | 22  25 ( 24 ) | 24  15 ( 20 ) | 7  6 ( 7 ) |
|  |  | 150 | 174  176 ( 175 ) | 12  8 ( 10 ) | 16  25 ( 21 ) | 14  19 ( 17 ) | 6  6 ( 6 ) |
|  |  | 500 | 331  282 ( 307 ) | 11  11 ( 11 ) | 35  42 ( 39 ) | 17  20 ( 19 ) | 7  7 ( 7 ) |
|  |  | 1500 | 731  645 ( 688 ) | 9  14 ( 12 ) | 73  73 ( 73 ) | 42  46 ( 44 ) | 7  9 ( 8 ) |
|  |  | 5000 | 0 *  0 * ( 0 ) | 0 *  0 * ( 0 ) | 7 *  4 * ( 6 ) | 0 *  0 * ( 0 ) | 0 *  0 * ( 0 ) |
| S9 mix  (+) | | Negative control | 118  128 ( 123 ) | 15  15 ( 15 ) | 23  22 ( 23 ) | 23  30 ( 27 ) | 12  15 ( 14 ) |
|  |  | 15.0 | 123  142 ( 133 ) | 9  11 ( 10 ) | 19  26 ( 23 ) | 35  22 ( 29 ) | 21  19 ( 20 ) |
|  |  | 50.0 | 115  129 ( 122 ) | 9  9 ( 9 ) | 27  21 ( 24 ) | 24  23 ( 24 ) | 24  24 ( 24 ) |
|  |  | 150 | 122  128 ( 125 ) | 8  9 ( 9 ) | 20  26 ( 23 ) | 35  25 ( 30 ) | 15  13 ( 14 ) |
|  |  | 500 | 116  109 ( 113 ) | 8  14 ( 11 ) | 25  15 ( 20 ) | 32  29 ( 31 ) | 19  17 ( 18 ) |
|  |  | 1500 | 126  134 ( 130 ) | 11  12 ( 12 ) | 19  24 ( 22 ) | 35  36 ( 36 ) | 11  14 ( 13 ) |
|  |  | 5000 | 482  407 ( 445 ) | 15  17 ( 16 ) | 31  39 ( 35 ) | 50  66 ( 58 ) | 19  26 ( 23 ) |
|  | S9 mix  (-) | Positive controls | AF-2 | SA | AF-2 | AF-2 | 9AA |
|  |  | Dose (µg/plate) | 0.01 | 0.5 | 0.01 | 0.1 | 80 |
|  |  | Colonies/plate | 425  433 ( 429 ) | 635  696 ( 666 ) | 114  110 ( 112 ) | 521  525 ( 523 ) | 534  459 ( 497 ) |
|  | S9 mix  (+) | Positive controls | B[a]P | 2AA | 2AA | B[a]P | B[a]P |
|  |  | Dose (µg/plate) | 5 | 2 | 10 | 5 | 5 |
|  |  | Colonies/plate | 1393  1319 ( 1356 ) | 503  504 ( 504 ) | 693  801 ( 747 ) | 435  439 ( 437 ) | 184  152 ( 168 ) |

( ), the average number of colonies. Negative control, DMSO

AF-2, 2-(2-Furyl)-3-(5-nitro-2-furyl)acrylamide; SA, Sodium azide; 9AA, 9-Aminoacridine; B[a]P, Benzo[a]pyrene; 2AA, 2-Aminoanthracene

*, Growth inhibition

| Metabolic activation | | Dose  (µg/plate) | Colonies/plate | | | | |
| --- | --- | --- | --- | --- | --- | --- | --- |
|  |  |  | Base substitution | | | Frameshift | |
|  |  |  | TA100 | TA1535 | WP2 *uvrA* | TA98 | TA1537 |
| S9 mix  (-) | | Negative control | 117  108 ( 113 ) | 20  17 ( 19 ) | 28  34 ( 31 ) | 17  25 ( 21 ) | 10  7 ( 9 ) |
|  |  | 156 | 167  135 ( 151 ) | 22  11 ( 17 ) | 28  31 ( 30 ) | 23  19 ( 21 ) | 5  9 ( 7 ) |
|  |  | 313 | 221  192 ( 207 ) | 18  17 ( 18 ) | 34  33 ( 34 ) | 24  25 ( 25 ) | 7  4 ( 6 ) |
|  |  | 625 | 356  342 ( 349 ) | 23  24 ( 24 ) | 47  46 ( 47 ) | 26  18 ( 22 ) | 6  9 ( 8 ) |
|  |  | 1250 | 565  651 ( 608 ) | 24  20 ( 22 ) | 61  67 ( 64 ) | 43  29 ( 36 ) | 5  3 ( 4 ) |
|  |  | 2500 | 1079  1122 ( 1101 ) | 22  22 ( 22 ) | 88  89 ( 89 ) | 50  53 ( 52 ) | 3  1 ( 2 ) |
|  |  | 5000 | 0 *  0 * ( 0 ) | 0 *  0 * ( 0 ) | 0 *  0 * ( 0 ) | 0 *  0 * ( 0 ) | 0 *  0 * ( 0 ) |
| S9 mix  (+) | | Negative control | 137  139 ( 138 ) | 20  18 ( 19 ) | 26  37 ( 32 ) | 24  23 ( 24 ) | 16  13 ( 15 ) |
|  |  | 313 | 133  132 ( 133 ) | 17  19 ( 18 ) | 22  25 ( 24 ) | 28  23 ( 26 ) | 19  15 ( 17 ) |
|  |  | 625 | 148  114 ( 131 ) | 10  22 ( 16 ) | 21  24 ( 23 ) | 21  26 ( 24 ) | 14  18 ( 16 ) |
|  |  | 1250 | 133  129 ( 131 ) | 17  19 ( 18 ) | 29  18 ( 24 ) | 29  26 ( 28 ) | 15  12 ( 14 ) |
|  |  | 2500 | 197  171 ( 184 ) | 18  20 ( 19 ) | 27  33 ( 30 ) | 31  39 ( 35 ) | 17  12 ( 15 ) |
|  |  | 5000 | 619  561 ( 590 ) | 22  11 ( 17 ) | 21  40 ( 31 ) | 68  68 ( 68 ) | 18  10 ( 14 ) |
|  | S9 mix  (-) | Positive controls | AF-2 | SA | AF-2 | AF-2 | 9AA |
|  |  | Dose (µg/plate) | 0.01 | 0.5 | 0.01 | 0.1 | 80 |
|  |  | Colonies/plate | 437  438 ( 438 ) | 600  638 ( 619 ) | 114  108 ( 111 ) | 564  574 ( 569 ) | 485  621 ( 553 ) |
|  | S9 mix  (+) | Positive controls | B[a]P | 2AA | 2AA | B[a]P | B[a]P |
|  |  | Dose (µg/plate) | 5 | 2 | 10 | 5 | 5 |
|  |  | Colonies/plate | 1323  1329 ( 1326 ) | 478  518 ( 498 ) | 766  822 ( 794 ) | 446  473 ( 460 ) | 168  152 ( 160 ) |

**Appendix VIIb:** **furfuryl formate (Exp. 2)**

( ), the average number of colonies. Negative control, DMSO

AF-2, 2-(2-Furyl)-3-(5-nitro-2-furyl)acrylamide; SA, Sodium azide; 9AA, 9-Aminoacridine; B[a]P, Benzo[a]pyrene; 2AA, 2-Aminoanthracene

*, Growth inhibition

**Appendix VIIc:** **furfuryl formate (Relative Activity Value; RAV)**

|  | Strain | S9 mix (-) | | S9 mix (+) | |
| --- | --- | --- | --- | --- | --- |
|  |  | RAV* | Dose  (μg/plate) | RAV* | Dose  (μg/plate) |
| Exp. 1  . | TA100 | 374 | 500 | 64 | 5000 |
|  |  | 379 | 1500 |  |  |
|  | WP2 *uvrA* | 35 | 1500 | － | － |
|  | TA98 | 18 | 1500 | 6 | 5000 |
| Exp. 2 | TA100 | 378 | 625 | 90 | 5000 |
|  |  | 396 | 1250 |  |  |
|  |  | 395 | 2500 |  |  |
|  | WP2 *uvrA* | 26 | 1250 | － | － |
|  |  | 23 | 2500 |  |  |
|  | TA98 | 12 | 2500 | 9 | 5000 |

*: Colonies/mg

| Metaboliｃ activation | | Dose  (µg/plate) | Colonies/plate | | | | |
| --- | --- | --- | --- | --- | --- | --- | --- |
|  |  |  | Base substitution | | | Frameshift | |
|  |  |  | TA100 | TA1535 | WP2 *uvrA* | TA98 | TA1537 |
| S9 mix  (-) | | Negative control | 102  110 ( 106 ) | 10  12 ( 11 ) | 17  32 ( 25 ) | 18  17 ( 18 ) | 7  7 ( 7 ) |
|  |  | 15.0 | 131  141 ( 136 ) | 9  6 ( 8 ) | 18  23 ( 21 ) | 17  18 ( 18 ) | 11  9 ( 10 ) |
|  |  | 50.0 | 126  106 ( 116 ) | 10  9 ( 10 ) | 21  18 ( 20 ) | 23  24 ( 24 ) | 14  9 ( 12 ) |
|  |  | 150 | 122  126 ( 124 ) | 11  10 ( 11 ) | 20  20 ( 20 ) | 14  23 ( 19 ) | 4  5 ( 5 ) |
|  |  | 500 | 172  153 ( 163 ) | 14  6 ( 10 ) | 27  24 ( 26 ) | 25  17 ( 21 ) | 11  9 ( 10 ) |
|  |  | 1500 | 214  220 ( 217 ) | 16  13 ( 15 ) | 38  44 ( 41 ) | 18  23 ( 21 ) | 11  6 ( 9 ) |
|  |  | 5000 † | 0 *  0 * ( 0 ) | 0 *  0 * ( 0 ) | 44 *  45 * ( 45 ) | 0 *  0 * ( 0 ) | 0 *  0 * ( 0 ) |
| S9 mix  (+) | | Negative control | 127  99 ( 113 ) | 15  8 ( 12 ) | 19  21 ( 20 ) | 23  18 ( 21 ) | 12  13 ( 13 ) |
|  |  | 15.0 | 126  137 ( 132 ) | 8  10 ( 9 ) | 31  13 ( 22 ) | 36  16 ( 26 ) | 19  15 ( 17 ) |
|  |  | 50.0 | 151  133 ( 142 ) | 11  14 ( 13 ) | 22  31 ( 27 ) | 30  22 ( 26 ) | 7  20 ( 14 ) |
|  |  | 150 | 123  165 ( 144 ) | 10  12 ( 11 ) | 24  30 ( 27 ) | 21  14 ( 18 ) | 6  13 ( 10 ) |
|  |  | 500 | 158  138 ( 148 ) | 17  10 ( 14 ) | 33  21 ( 27 ) | 25  23 ( 24 ) | 16  19 ( 18 ) |
|  |  | 1500 | 192  173 ( 183 ) | 14  10 ( 12 ) | 40  40 ( 40 ) | 24  32 ( 28 ) | 12  18 ( 15 ) |
|  |  | 5000 | 0 *  0 * ( 0 ) | 0 *  3 * ( 2 ) | 48 *  15 * ( 32 ) | 10 *  10 * ( 10 ) | 0 *  0 * ( 0 ) |
|  | S9 mix  (-) | Positive controls | AF-2 | SA | AF-2 | AF-2 | 9AA |
|  |  | Dose (µg/plate) | 0.01 | 0.5 | 0.01 | 0.1 | 80 |
|  |  | Colonies/plate | 469  428 ( 449 ) | 543  632 ( 588 ) | 125  126 ( 126 ) | 552  535 ( 544 ) | 533  434 ( 484 ) |
|  | S9 mix  (+) | Positive controls | B[a]P | 2AA | 2AA | B[a]P | B[a]P |
|  |  | Dose (µg/plate) | 5 | 2 | 10 | 5 | 5 |
|  |  | Colonies/plate | 1346  1246 ( 1296 ) | 555  488 ( 522 ) | 721  758 ( 740 ) | 487  406 ( 447 ) | 163  176 ( 170 ) |

**Appendix VIIIa: methyl beta-phenylglycidate (Exp. 1)**

( ), the average number of colonies. Negative control, DMSO

AF-2, 2-(2-Furyl)-3-(5-nitro-2-furyl)acrylamide; SA, Sodium azide; 9AA, 9-Aminoacridine; B[a]P, Benzo[a]pyrene; 2AA, 2-Aminoanthracene

†, Precipitation

*, Growth inhibition

| Metabolic activation | | Dose  (µg/plate) | Colonies/plate | | | | |
| --- | --- | --- | --- | --- | --- | --- | --- |
|  |  |  | Base substitution | | | Frameshift | |
|  |  |  | TA100 | TA1535 | WP2 *uvrA* | TA98 | TA1537 |
| S9 mix  (-) | | Negative control | 100  108 ( 104 ) | 15  15 ( 15 ) | 19  17 ( 18 ) | 22  14 ( 18 ) | 8  6 ( 7 ) |
|  |  | 156 | 119  129 ( 124 ) | 15  24 ( 20 ) | 18  24 ( 21 ) | 18  33 ( 26 ) | 6  6 ( 6 ) |
|  |  | 313 | 118  136 ( 127 ) | 12  19 ( 16 ) | 36  27 ( 32 ) | 20  24 ( 22 ) | 11  11 ( 11 ) |
|  |  | 625 | 143  169 ( 156 ) | 19  15 ( 17 ) | 30  29 ( 30 ) | 25  26 ( 26 ) | 10  4 ( 7 ) |
|  |  | 1250 | 210  208 ( 209 ) | 15  15 ( 15 ) | 35  33 ( 34 ) | 27  23 ( 25 ) | 9  4 ( 7 ) |
|  |  | 2500 | 260 *  291 * ( 276 ) | 13 *  17 * ( 15 ) | 53  40 ( 47 ) | 21  21 ( 21 ) | 6  6 ( 6 ) |
|  |  | 5000 † | 0 *  0 * ( 0 ) | 2 *  0 * ( 1 ) | 67 *  67 * ( 67 ) | 8 *  14 * ( 11 ) | 1 *  9 * ( 5 ) |
| S9 mix  (+) | | Negative control | 136  130 ( 133 ) | 20  20 ( 20 ) | 22  26 ( 24 ) | 34  27 ( 31 ) | 10  21 ( 16 ) |
|  |  | 156 | 144  128 ( 136 ) | 21  16 ( 19 ) | 32  33 ( 33 ) | 27  31 ( 29 ) | 16  10 ( 13 ) |
|  |  | 313 | 110  156 ( 133 ) | 20  24 ( 22 ) | 25  26 ( 26 ) | 30  25 ( 28 ) | 10  16 ( 13 ) |
|  |  | 625 | 139  149 ( 144 ) | 21  17 ( 19 ) | 41  31 ( 36 ) | 30  26 ( 28 ) | 12  14 ( 13 ) |
|  |  | 1250 | 169  188 ( 179 ) | 14  17 ( 16 ) | 43  47 ( 45 ) | 32  29 ( 31 ) | 15  17 ( 16 ) |
|  |  | 2500 | 199  186 ( 193 ) | 22  20 ( 21 ) | 75  45 ( 60 ) | 24  23 ( 24 ) | 13  16 ( 15 ) |
|  |  | 5000 | 0 *  0 * ( 0 ) | 0 *  0 * ( 0 ) | 42 *  22 * ( 32 ) | 0 *  0 * ( 0 ) | 0 *  0 * ( 0 ) |
|  | S9 mix  (-) | Positive controls | AF-2 | SA | AF-2 | AF-2 | 9AA |
|  |  | Dose (µg/plate) | 0.01 | 0.5 | 0.01 | 0.1 | 80 |
|  |  | Colonies/plate | 440  466 ( 453 ) | 631  584 ( 608 ) | 102  104 ( 103 ) | 580  552 ( 566 ) | 545  535 ( 540 ) |
|  | S9 mix  (+) | Positive controls | B[a]P | 2AA | 2AA | B[a]P | B[a]P |
|  |  | Dose (µg/plate) | 5 | 2 | 10 | 5 | 5 |
|  |  | Colonies/plate | 1300  1284 ( 1292 ) | 514  513 ( 514 ) | 733  812 ( 773 ) | 406  452 ( 429 ) | 179  169 ( 174 ) |

**Appendix VIIIb: methyl beta-phenylglycidate (Exp. 2)**

( ), the average number of colonies. Negative control, DMSO

AF-2, 2-(2-Furyl)-3-(5-nitro-2-furyl)acrylamide; SA, Sodium azide; 9AA, 9-Aminoacridine; B[a]P, Benzo[a]pyrene; 2AA, 2-Aminoanthracene

†, Precipitation

*, Growth inhibition

| Metabolic activation | | Dose  (µg/plate) | Colonies/plate | | | |
| --- | --- | --- | --- | --- | --- | --- |
|  |  |  | Base substitution | | | |
|  |  |  | WP2 *uvrA* | | | |
|  | | Negative control | 33 |  |  |  |
|  | |  | 34 | ( | 34 | ) |
|  | | 1000 | 46 |  |  |  |
|  | |  | 38 | ( | 42 | ) |
|  | | 2000 | 68 |  |  |  |
| S9 mix | |  | 41 | ( | 55 | ) |
|  | | 2500 | 55 |  |  |  |
|  | |  | 65 | ( | 60 | ) |
| (-) | | 3000 | 71 * |  |  |  |
|  | |  | 66 * | ( | 69 | ) |
|  | | 4000 | 44 * |  |  |  |
|  | |  | 48 * | ( | 46 | ) |
|  | | 5000 † | 49 * |  |  |  |
|  | |  | 57 * | ( | 53 | ) |
|  | S9 mix  (-) | Positive controls | AF-2 | | | |
|  |  | Dose (µg/plate) | 0.01 | | | |
|  |  | Colonies/plate | 126  134 ( 130 ) | | | |

**Appendix VIIIc: methyl beta-phenylglycidate (Exp. 3)**

( ), the average number of colonies. Negative control,DMSO

AF-2, 2-(2-Furyl)-3-(5-nitro-2-furyl)acrylamide

†, Precipitation

*, Growth inhibition

**Appendix VIIId: methyl beta-phenylglycidate (Relative Activity Value; RAV)**

|  | Strain | S9 mix (-) | | S9 mix (+) | |
| --- | --- | --- | --- | --- | --- |
|  |  | RAV* | Dose  (μg/plate) | RAV* | Dose  (μg/plate) |
| Exp. 1 | TA100 | 74 | 1500 | － | － |
|  | WP2 *uvrA* | － | － | 13 | 1500 |
| Exp. 2 | TA100 | 84 | 1250 | － | － |
|  |  | 69 | 2500 |  |  |
|  | WP2 *uvrA* | 12 | 2500 | 14 | 2500 |
|  |  | 10 | 5000 |  |  |
| Exp. 3 | WP2 *uvrA* | 12 | 3000 | － | － |

*: Colonies/mg

| Metabolic activation | | Dose  (µg/plate) | Colonies/plate | | | | |
| --- | --- | --- | --- | --- | --- | --- | --- |
|  |  |  | Base substitution | | | Frameshift | |
|  |  |  | TA100 | TA1535 | WP2 *uvrA* | TA98 | TA1537 |
| S9 mix  (-) | | Negative control | 95  104 ( 100 ) | 9  9 ( 9 ) | 31  34 ( 33 ) | 15  14 ( 15 ) | 18  12 ( 15 ) |
|  |  | 5.00 | 126  115 ( 121 ) | 11  10 ( 11 ) | 41  35 ( 38 ) | 19  15 ( 17 ) | 14  16 ( 15 ) |
|  |  | 15.0 | 127  100 ( 114 ) | 10  13 ( 12 ) | 34  29 ( 32 ) | 20  24 ( 22 ) | 20  7 ( 14 ) |
|  |  | 50.0 | 106  120 ( 113 ) | 8  7 ( 8 ) | 23  38 ( 31 ) | 18  14 ( 16 ) | 21  10 ( 16 ) |
|  |  | 150 | 91  109 ( 100 ) | 11  6 ( 9 ) | 23  42 ( 33 ) | 16  11 ( 14 ) | 10  21 ( 16 ) |
|  |  | 500 | 94  98 ( 96 ) | 9  7 ( 8 ) | 38  27 ( 33 ) | 18  15 ( 17 ) | 13  15 ( 14 ) |
|  |  | 1500 | 79  67 ( 73 ) | 7  8 ( 8 ) | 33  28 ( 31 ) | 16  15 ( 16 ) | 12  7 ( 10 ) |
|  |  | 5000 | 0 *  0 * ( 0 ) | 0 *  0 * ( 0 ) | 0 *  0 * ( 0 ) | 0 *  0 * ( 0 ) | 0 *  0 * ( 0 ) |
| S9 mix  (+) | | Negative control | 119  140 ( 130 ) | 8  6 ( 7 ) | 41  40 ( 41 ) | 21  31 ( 26 ) | 19  19 ( 19 ) |
|  |  | 5.00 | 383  326 ( 355 ) | 13  11 ( 12 ) | 76  81 ( 79 ) | 39  53 ( 46 ) | 33  27 ( 30 ) |
|  |  | 15.0 | 734  710 ( 722 ) | 31  33 ( 32 ) | 147  156 ( 152 ) | 75  74 ( 75 ) | 40  38 ( 39 ) |
|  |  | 50.0 | 900  982 ( 941 ) | 31  38 ( 35 ) | 287  267 ( 277 ) | 90  93 ( 92 ) | 64  60 ( 62 ) |
|  |  | 150 | 1028  962 ( 995 ) | 54  38 ( 46 ) | 342  339 ( 341 ) | 77  81 ( 79 ) | 67  80 ( 74 ) |
|  |  | 500 | 898  823 ( 861 ) | 35  32 ( 34 ) | 287  258 ( 273 ) | 79  67 ( 73 ) | 66  56 ( 61 ) |
|  |  | 1500 | 666  660 ( 663 ) | 44  37 ( 41 ) | 174  143 ( 159 ) | 45  54 ( 50 ) | 40  36 ( 38 ) |
|  |  | 5000 | 0 *  0 * ( 0 ) | 0 *  0 * ( 0 ) | 0 *  0 * ( 0 ) | 0 *  0 * ( 0 ) | 0 *  0 * ( 0 ) |
|  | S9 mix  (-) | Positive controls | AF-2 | SA | AF-2 | AF-2 | 9AA |
|  |  | Dose (µg/plate) | 0.01 | 0.5 | 0.01 | 0.1 | 80 |
|  |  | Colonies/plate | 443  417 ( 430 ) | 553  547 ( 550 ) | 128  127 ( 128 ) | 531  561 ( 546 ) | 307  358 ( 333 ) |
|  | S9 mix  (+) | Positive controls | B[a]P | 2AA | 2AA | B[a]P | B[a]P |
|  |  | Dose (µg/plate) | 5 | 2 | 10 | 5 | 5 |
|  |  | Colonies/plate | 1301  1297 ( 1299 ) | 481  499 ( 490 ) | 645  786 ( 716 ) | 455  402 ( 429 ) | 170  165 ( 168 ) |

**Appendix IXa: 6-methoxyquinoline (Exp. 1)**

( ), the average number of colonies. Negative control, DMSO

AF-2, 2-(2-Furyl)-3-(5-nitro-2-furyl)acrylamide; SA, Sodium azide; 9AA, 9-Aminoacridine; B[a]P, Benzo[a]pyrene; 2AA, 2 Aminoanthracene

*, Growth inhibition

| Metabolic activation | | Dose  (µg/plate) | Colonies/plate | | | | |
| --- | --- | --- | --- | --- | --- | --- | --- |
|  |  |  | Base substitution | | | Frame shift | |
|  |  |  | TA100 | TA1535 | WP2 *uvrA* | TA98 | TA1537 |
| S9 mix  (-) | | Negative control | 110  104 ( 107 ) | 11  12 ( 12 ) | 23  28 ( 26 ) | 11  14 ( 13 ) | 13  10 ( 12 ) |
|  |  | 156 | 86  97 ( 92 ) | 9  15 ( 12 ) | 33  31 ( 32 ) | 13  17 ( 15 ) | 10  16 ( 13 ) |
|  |  | 313 | 113  94 ( 104 ) | 5  10 ( 8 ) | 21  17 ( 19 ) | 13  23 ( 18 ) | 16  14 ( 15 ) |
|  |  | 625 | 82  87 ( 85 ) | 7  9 ( 8 ) | 14  17 ( 16 ) | 15  13 ( 14 ) | 19  14 ( 17 ) |
|  |  | 1250 | 88  83 ( 86 ) | 10  5 ( 8 ) | 15  22 ( 19 ) | 17  18 ( 18 ) | 16  14 ( 15 ) |
|  |  | 2500 | 56 *  38 * ( 47 ) | 8 *  7 * ( 8 ) | 18  17 ( 18 ) | 8  10 ( 9 ) | 9  7 ( 8 ) |
|  |  | 5000 | 0 *  0 * ( 0 ) | 0 *  0 * ( 0 ) | 0 *  0 * ( 0 ) | 0 *  0 * ( 0 ) | 0 *  0 * ( 0 ) |
| S9 mix  (+) | | Negative control | 105  118 ( 112 ) | 13  9 ( 11 ) | 17  28 ( 23 ) | 23  18 ( 21 ) | 28  24 ( 26 ) |
|  |  | 1.22 | 211  169 ( 190 ) | NT | NT | NT | NT |
|  |  | 2.44 | 231  226 ( 229 ) | NT | NT | NT | NT |
|  |  | 4.88 | 345  375 ( 360 ) | 13  22 ( 18 ) | 77  68 ( 73 ) | 31  49 ( 40 ) | 36  33 ( 35 ) |
|  |  | 9.77 | 630  594 ( 612 ) | 12  29 ( 21 ) | 120  100 ( 110 ) | 71  76 ( 74 ) | 34  38 ( 36 ) |
|  |  | 19.5 | 817  841 ( 829 ) | 33  24 ( 29 ) | 172  193 ( 183 ) | 87  88 ( 88 ) | 50  58 ( 54 ) |
|  |  | 39.1 | 790  906 ( 848 ) | 24  37 ( 31 ) | 212  254 ( 233 ) | 77  85 ( 81 ) | 65  54 ( 60 ) |
|  |  | 78.1 | 989  899 ( 944 ) | 30  50 ( 40 ) | 311  295 ( 303 ) | 89  77 ( 83 ) | 51  82 ( 67 ) |
|  |  | 156 | 857  856 ( 857 ) | 48  38 ( 43 ) | 363  291 ( 327 ) | 92  97 ( 95 ) | 62  70 ( 66 ) |
|  |  | 313 | 883  916 ( 900 ) | 37  39 ( 38 ) | 304  248 ( 276 ) | 86  64 ( 75 ) | 61  57 ( 59 ) |
|  |  | 1250 | 690  700 ( 695 ) | 36  52 ( 44 ) | 115  139 ( 127 ) | 52  51 ( 52 ) | 53  50 ( 52 ) |
|  |  | 5000 | 0 *  0 * ( 0 ) | 0 *  0 * ( 0 ) | 0 *  0 * ( 0 ) | 0 *  0 * ( 0 ) | 0 *  0 * ( 0 ) |
|  | S9 mix  (-) | Positive controls | AF-2 | SA | AF-2 | AF-2 | 9AA |
|  |  | Dose (µg/plate) | 0.01 | 0.5 | 0.01 | 0.1 | 80 |
|  |  | Colonies/plate | 467  460 ( 464 ) | 532  603 ( 568 ) | 124  109 ( 117 ) | 613  603 ( 608 ) | 532  383 ( 458 ) |
|  | S9 mix  (+) | Positive controls | B[a]P | 2AA | 2AA | B[a]P | B[a]P |
|  |  | Dose (µg/plate) | 5 | 2 | 10 | 5 | 5 |
|  |  | Colonies/plate | 1459  1372 ( 1416 ) | 475  476 ( 476 ) | 703  780 ( 742 ) | 456  428 ( 442 ) | 199  158 ( 179 ) |

**Appendix IXb: 6-methoxyquinoline (Exp. 2)**

( )

( ), the average number of colonies. Negative control, DMSO

AF-2, 2-(2-Furyl)-3-(5-nitro-2-furyl)acrylamide; SA, Sodium azide; 9AA, 9-Aminoacridine; B[a]P, Benzo[a]pyrene; 2AA, 2-Aminoanthracene

*, Growth inhibition,; NT, Not tested

**Appendix IXc: 6-methoxyquinoline**

|  | Strain | S9 mix (+) | |
| --- | --- | --- | --- |
|  |  | RAV* | Dose  (μg/plate) |
| Exp. 1 | TA100 | 45000 | 5.00 |
|  |  | 39467 | 15.0 |
|  |  | 16220 | 50.0 |
|  |  | 5767 | 150 |
|  |  | 1462 | 500 |
|  |  | 355 | 1500 |
|  | TA1535 | 1667 | 15.0 |
|  |  | 560 | 50.0 |
|  |  | 260 | 150 |
|  |  | 54 | 500 |
|  |  | 23 | 1500 |
|  | WP2 *uvrA* | 7400 | 15.0 |
|  |  | 4720 | 50.0 |
|  |  | 2000 | 150 |
|  |  | 464 | 500 |
|  |  | 79 | 1500 |
|  | TA98 | 3267 | 15.0 |
|  |  | 1320 | 50.0 |
|  |  | 353 | 150 |
|  |  | 94 | 500 |
|  | TA1537 | 1333 | 15.0 |
|  |  | 860 | 50.0 |
|  |  | 367 | 150 |
|  |  | 84 | 500 |
|  |  | 13 | 1500 |

**(Relative Activity Value; RAV)**

*: Colonies/mg

|  | Strain | S9 mix (+) | |
| --- | --- | --- | --- |
|  |  | RAV* | Dose  (μg/plate) |
| Exp. 2 | TA100 | 47951 | 2.44 |
|  |  | 50820 | 4.88 |
|  |  | 51177 | 9.77 |
|  |  | 36769 | 19.5 |
|  |  | 18824 | 39.1 |
|  |  | 10653 | 78.1 |
|  |  | 4776 | 156 |
|  |  | 2518 | 313 |
|  |  | 466 | 1250 |
|  | TA1535 | 923 | 19.5 |
|  |  | 512 | 39.1 |
|  |  | 371 | 78.1 |
|  |  | 205 | 156 |
|  |  | 86 | 313 |
|  |  | 26 | 1250 |
|  | WP2 *uvrA* | 10246 | 4.88 |
|  |  | 8905 | 9.77 |
|  |  | 8205 | 19.5 |
|  |  | 5371 | 39.1 |
|  |  | 3585 | 78.1 |
|  |  | 1949 | 156 |
|  |  | 808 | 313 |
|  |  | 83 | 1250 |
|  | TA98 | 5425 | 9.77 |
|  |  | 3436 | 19.5 |
|  |  | 1535 | 39.1 |
|  |  | 794 | 78.1 |
|  |  | 474 | 156 |
|  |  | 173 | 313 |
|  |  | 25 | 1250 |
|  | TA1537 | 1436 | 19.5 |
|  |  | 870 | 39.1 |
|  |  | 525 | 78.1 |
|  |  | 256 | 156 |
|  |  | 105 | 313 |
|  |  | 21 | 1250 |

*: Col

*Colonies/mg

**Appendix Xa: 2-methylquinoline (Exp. 1a)**

| Metabolic activation | | Dose (µg/plate) | Colonies/plate | | | | |
| --- | --- | --- | --- | --- | --- | --- | --- |
|  |  |  | Base substitution | | | Frameshift | |
|  |  |  | TA100 | TA1535 | WP2 *uvrA* | TA98 | TA1537 |
| -S9 mix | | Negative control | 121 (114)  106 | 12 ( 15)  17 | 30 ( 29)  28 | 18 ( 21)  24 | 9 ( 8)  7 |
|  |  | 0.305 | 119 (118)  116 | 11 ( 15)  18 | 40 ( 31)  22 | 20 ( 20)  20 | 5 ( 6)  7 |
|  |  | 1.22 | 108 (117)  126 | 12 ( 15)  17 | 28 ( 29)  29 | 19 ( 22)  24 | 9 ( 8)  6 |
|  |  | 4.88 | 114 (114)  114 | 9 ( 9)  8 | 22 ( 25)  27 | 19 ( 22)  24 | 8 ( 8)  8 |
|  |  | 19.5 | 104 (104)  104 | 12 ( 15)  18 | 28 ( 25)  21 | 18 ( 22)  25 | 6 ( 6)  6 |
|  |  | 78.1 | 109 (108)  106 | 10 ( 12)  13 | 23 ( 24)  25 | 17 ( 21)  24 | 8 ( 10)  11 |
|  |  | 313 | 116 (109)  101 | 17 ( 14)  10 | 25 ( 23)  21 | 20 ( 19)  17 | 7 ( 9)  10 |
|  |  | 1250 | 108 (109)  109 | 11 ( 11)  10 | 34 ( 30)  25 | 19 ( 22)  24 | 5 ( 6)  7 |
|  |  | 5000 | 0 * ( 0)  0 * | 0 * ( 0)  0 * | 10 * ( 9)  8 * | 0 * ( 0)  0 * | 0 * ( 0)  0 * |
|  | S9 mix  (-) | Positive controls | AF-2 | AZI | AF-2 | AF-2 | 9AA |
|  |  | Dose (µg/plate) | 0.01 | 0.5 | 0.01 | 0.1 | 80.0 |
|  |  | Colonies/plate | 609 (605)  600 | 638 (625)  612 | 179 (157)  134 | 449 (472)  495 | 234 (241)  248 |

( ), the average number of colonies. Negative control, DMSO

AF-2: 2-(2-Furyl)-3-(5-nitro-2-furyl)acrylamide, AZI: Sodium azide, 9AA: 9-Aminoacridine

*, Growth inhibition

**Appendix Xb: 2-methylquinoline (Exp. 1b)**

| Metabolic activation | | Dose (µg/plate) | Colonies/plate | | | | |
| --- | --- | --- | --- | --- | --- | --- | --- |
|  |  |  | Base substitution | | | Frameshift | |
|  |  |  | TA100 | TA1535 | WP2 *uvrA* | TA98 | TA1537 |
| + S9 mix | | Negative control | 131 (132)  132 | 18 ( 17)  15 | 23 ( 27)  30 | 37 ( 32)  27 | 15 ( 13)  11 |
|  |  | 0.305 | 115 (110)  105 | 12 ( 13)  14 | 38 ( 34)  30 | 22 ( 24)  26 | 11 ( 11)  11 |
|  |  | 1.22 | 106 (125)  144 | 16 ( 15)  13 | 33 ( 25)  16 | 33 ( 35)  37 | 10 ( 9)  8 |
|  |  | 4.88 | 110 (106)  102 | 10 ( 14)  17 | 28 ( 34)  39 | 28 ( 28)  28 | 11 ( 14)  16 |
|  |  | 19.5 | 124 (136)  148 | 9 ( 11)  13 | 20 ( 24)  28 | 26 ( 29)  31 | 8 ( 9)  10 |
|  |  | 78.1 | 155 (147)  138 | 14 ( 11)  7 | 24 ( 25)  25 | 37 ( 38)  38 | 11 ( 11)  10 |
|  |  | 313 | 188 (192)  195 | 14 ( 13)  12 | 30 ( 36)  41 | 27 ( 26)  25 | 16 ( 16)  16 |
|  |  | 1250 | 261 (261)  260 | 17 ( 13)  9 | 31 ( 31)  31 | 30 ( 25)  19 | 11 ( 11)  10 |
|  |  | 5000 | 0 * ( 0)  0 * | 0 * ( 0)  0 * | 0 * ( 0)  0 * | 0 * ( 0)  0 * | 0 * ( 0)  0 * |
|  | S9 mix  (+) | Positive controls | 2AA | 2AA | 2AA | 2AA | 2AA |
|  |  | Dose (µg/plate) | 1.0 | 2.0 | 10.0 | 0.5 | 2.0 |
|  |  | Colonies/plate | 1341 (1350)  1359 | 541 (517)  493 | 1180 (1234)  1288 | 646 (633)  619 | 240 (235)  229 |

( ), the average number of colonies. Negative control, DMSO

2AA: 2-Aminoanthracene

*, Growth inhibition

**Appendix Xc: 2-methylquinoline (Exp. 2)**

| Metabolic activation | | Dose (µg/plate) | Colonies/plate | | | | |
| --- | --- | --- | --- | --- | --- | --- | --- |
|  |  |  | Base substitution | | | Frameshift | |
|  |  |  | TA100 | TA1535 | WP2 *uvrA* | TA98 | TA1537 |
| -S9 mix | | Negative control | 108 (112)  115 | 17 ( 19)  20 | 22 ( 22)  22 | 24 ( 23)  21 | 8 ( 7)  6 |
|  |  | 156 | 105 (101)  96 | 11 ( 16)  20 | 20 ( 21)  22 | 21 ( 21)  21 | 9 ( 8)  6 |
|  |  | 313 | 116 ( 97)  78 | 21 ( 23)  25 | 14 ( 19)  24 | 12 ( 19)  26 | 10 ( 10)  9 |
|  |  | 625 | 97 ( 95)  93 | 19 ( 21)  22 | 24 ( 21)  18 | 15 ( 14)  13 | 8 ( 9)  10 |
|  |  | 1250 | 73 ( 71)  69 | 12 ( 18)  23 | 11 ( 12)  13 | 17 ( 18)  18 | 10 ( 8)  5 |
|  |  | 2500 | 49 * ( 46)  43 * | 10 * ( 9)  8 * | 9 * ( 9)  8 * | 5 * ( 6)  6 * | 6 * ( 5)  3 * |
|  |  | 5000 | 0 * ( 0)  0 * | 2 * ( 5)  7 * | 2 * ( 2)  1 * | 0 * ( 0)  0 * | 0 * ( 0)  0 * |
| + S9 mix | | Negative control | 133 (135)  137 | 20 ( 20)  20 | 26 ( 32)  38 | 34 ( 33)  32 | 12 ( 14)  15 |
|  |  | 156 | 206 (202)  198 | 17 ( 21)  25 | 35 ( 32)  29 | 37 ( 41)  44 | 13 ( 12)  11 |
|  |  | 313 | 283 (280)  276 | 17 ( 20)  22 | 30 ( 29)  28 | 29 ( 35)  40 | 15 ( 16)  17 |
|  |  | 625 | 345 (312)  279 | 14 ( 17)  19 | 30 ( 31)  32 | 20 ( 23)  26 | 18 ( 13)  8 |
|  |  | 1250 | 271 (280)  289 | 11 ( 15)  19 | 24 ( 27)  29 | 36 ( 34)  32 | 11 ( 13)  15 |
|  |  | 2500 | 74 * ( 73)  71 * | 8 * ( 8)  7 * | 18 * ( 18)  17 * | 16 * ( 14)  11 * | 12 * ( 9)  5 * |
|  |  | 5000 | 0 * ( 0)  0 * | 0 * ( 0)  0 * | 4 * ( 4)  3 * | 0 * ( 0)  0 * | 0 * ( 0)  0 * |
|  | S9 mix  (-) | Positive controls | AF-2 | AZI | AF-2 | AF-2 | 9AA |
|  |  | Dose (µg/plate) | 0.01 | 0.5 | 0.01 | 0.1 | 80.0 |
|  |  | Colonies/plate | 518 (515)  512 | 656 (643)  629 | 164 (144)  123 | 392 (406)  420 | 228 (253)  277 |
|  | S9 mix  (+) | Positive controls | 2AA | 2AA | 2AA | 2AA | 2AA |
|  |  | Dose (µg/plate) | 1.0 | 2.0 | 10.0 | 0.5 | 2.0 |
|  |  | Colonies/plate | 1015 (1065)  1114 | 538 (521)  504 | 1452 (1520)  1587 | 511 (505)  498 | 188 (194)  199 |

( ), the average number of colonies. Negative control, DMSO

AF-2: 2-(2-Furyl)-3-(5-nitro-2-furyl)acrylamide, AZI: Sodium azide, 9AA: 9-Aminoacridine, 2AA: 2-Aminoanthracene

*, Growth inhibition

**Appendix Xd: 2-methylquinoline (Exp. 3)**

| Metabolic activation | | Dose (µg/plate) | Colonies/plate |
| --- | --- | --- | --- |
|  |  |  | Base substitution |
|  |  |  | TA100 |
| -S9 mix | | Negative control | 140 (136)  132 |
| + S9 mix | | Negative control | 132 (133)  133 |
|  |  | 156 | 175 (207)  238 |
|  |  | 313 | 328 (322)  315 |
|  |  | 625 | 383 (396)  409 |
|  |  | 1250 | 267 (270)  273 |
|  |  | 2500 | 114 * (117)  120 * |
|  |  | 5000 | 0 * ( 0)  0 * |
|  | S9 mix  (-) | Positive controls | AF-2 |
|  |  | Dose (µg/plate) | 0.01 |
|  |  | Colonies/plate | 697 (680)  663 |
|  | S9 mix  (+) | Positive controls | 2AA |
|  |  | Dose (µg/plate) | 1.0 |
|  |  | Colonies/plate | 1432 (1459)  1485 |

( ), the average number of colonies. Negative control, DMSO

AF-2: 2-(2-Furyl)-3-(5-nitro-2-furyl)acrylamide 2AA: 2-Aminoanthracene

*, Growth inhibition

**Appendix Xe: 2-methylquinoline (Relative Activity Value; RAV)**

|  | Strain | S9 mix (-) | | S9 mix (+) | |
| --- | --- | --- | --- | --- | --- |
|  |  | RAV* | Dose  (μg/plate) | RAV* | Dose  (μg/plate) |
| Exp. 1  Exp. 3 | TA100 |  |  |  |  |
|  | TA1535 |  |  |  |  |
|  | WP2 *uvrA* |  |  |  |  |
|  | TA98 |  |  |  |  |
|  | TA1537 |  |  |  |  |
| Exp. 2  p. 2 | TA100 |  |  | 463 | 313 µg/plate |
|  | TA1535 |  |  |  |  |
|  | WP2 *uvrA* |  |  |  |  |
|  | TA98 |  |  |  |  |
|  | TA1537 |  |  |  |  |
| Exp. 3 | TA100 | ――― | ――― | 604 | 313 µg/plate |
|  |  |  |  |  |  |
|  |  |  |  |  |  |

*: Colonies/mg
